# Nitrogen remobilisation facilitates adventitious root formation on reversible dark-induced carbohydrate depletion in *Petunia hybrida*

**DOI:** 10.1186/s12870-016-0901-6

**Published:** 2016-10-10

**Authors:** Siegfried Zerche, Klaus-Thomas Haensch, Uwe Druege, Mohammad-Reza Hajirezaei

**Affiliations:** 1 Department of Plant Nutrition, Leibniz Institute of Vegetable & Ornamental Crops (IGZ), Kuehnhaeuser Str. 101, 99090 Erfurt, Germany; 2 Department of Plant Propagation, Leibniz Institute of Vegetable & Ornamental Crops (IGZ), Kuehnhaeuser Str. 101, 99090 Erfurt, Germany; 3 Leibniz Institute of Plant Genetics and Crop Plant Research (IPK), Molecular Plant Nutrition, Corrensstr. 3, 06466 Gatersleben, Germany

**Keywords:** Root primordium, Meristem, Root elongation, Nitrogen deficiency, Dark response, Carbohydrate depletion, Amino acids, Adventitious root formation

## Abstract

**Background:**

Adventitious root (AR) formation in axillary shoot tip cuttings is a crucial physiological process for ornamental propagation that is utilised in global production chains for young plants. In this process, the nitrogen and carbohydrate metabolisms of a cutting are regulated by its total nitrogen content (N_t_), dark exposure during transport and irradiance levels at distinct production sites and phases through a specific plasticity to readjust metabolite pools. Here, we examined how elevated N_t_ contents with a combined dark exposure of cuttings influence their internal N-pools including free amino acids and considered early anatomic events of AR formation as well as further root development in *Petunia hybrida* cuttings.

**Results:**

Enhanced N_t_ contents of unrooted cuttings resulted in elevated total free amino acid levels and in particular glutamate (glu) and glutamine (gln) in leaf and basal stem. N-allocation to mobile N-pools increased whereas the allocation to insoluble protein-N declined. A dark exposure of cuttings conserved initial N_t_ and nitrate-N, while it reduced insoluble protein-N and increased soluble protein, amino- and amide-N. The increase of amino acids mainly comprised asparagine (asn), aspartate (asp) and arginine (arg) in the leaves, with distinct tissue specific responses to an elevated N supply. Dark exposure induced an early transient rise of asp followed by a temporary increase of glu. A strong positive N effect of high N_t_ contents of cuttings on AR formation after 384 h was observed. Root meristematic cells developed at 72 h with a negligible difference for two N_t_ levels. After 168 h, an enhanced N_t_ accelerated AR formation and gave rise to first obvious fully developed roots while only meristems were formed with a low N_t_. However, dark exposure for 168 h promoted AR formation particularly in cuttings with a low N_t_ to such an extent so that the benefit of the enhanced N_t_ was almost compensated. Combined dark exposure and low N_t_ of cuttings strongly reduced shoot growth during AR formation.

**Conclusions:**

The results indicate that both enhanced N_t_ content and dark exposure of cuttings reinforced N signals and mobile N resources in the stem base facilitated by senescence-related proteolysis in leaves. Based on our results, a model of N mobilisation concomitant with carbohydrate depletion and its significance for AR formation is postulated.

**Electronic supplementary material:**

The online version of this article (doi:10.1186/s12870-016-0901-6) contains supplementary material, which is available to authorized users.

## Background

Adventitious root (AR) formation with high economic significance in horticulture, agriculture and forestry is a complex physiological process. The ornamental plant propagation relies on globalised chains for young plant production via rooting of cuttings ensuring an effective utilization of beneficial external and internal factors. The whole process includes three phases, axillary bud and shoot growth on donor plants (providing recurrent excision of mature shoot tips - i.e. cuttings), subsequent logistics (i.e. transport, storage) of cuttings and insertion of the cuttings into rooting media. During this process strong transcriptomic and metabolic changes occur with high importance of nitrogen availability, dark exposure and various irradiance levels. Thus, reciprocal regulations force adaptations in nitrogen and carbohydrate metabolism during phases of axillary bud and shoot growth, dark induced senescence of cuttings, stress recovery under diurnal light and AR formation in cuttings. It has already been shown that the level of nitrogen assimilation by donor plants changes nitrogen fluxes and rebalances the pools of carbohydrates and amino acids [1, 2]. Moreover, degradation and re-synthesis of proteins enable survival of rootless cuttings and are required for the regeneration of the missing root organs. Since AR formation relies on selective proteolysis and re-synthesis of proteins, the total nitrogen stock in the cuttings constitutes a key limiting factor [3, 4]. Interestingly, there are similarities and differences between AR formation and lateral roots [5, 6] especially for nitrogen deficiency and ethylene signalling and synthesis in planta. N deficiency stimulates lateral roots of sessile plants having already their intact root system. Then lateral root formation starts with highly cell-specific responses to external nitrogen signals that are directed towards nutrient-rich soil patches to ensure nutrient acquisition [7]. In contrast, excised axillary shoot tips (i.e. cuttings) such as petunia cuttings experience wounding and isolation and thus solely rely on shoot-born signals with specific transcriptome and metabolome responses [8–10]. When the vascular continuum collapses, auxin accumulates and induces AR formation in stem base tissue [11]. Primary auxin control of AR formation depends on secondary signals like nitric oxide, polyamines and ethylene [6, 12, 13]. Recently, an aminotransferase protein was reported to coordinate the biosynthesis of the hormones ethylene and auxin [14]. Further, auxin triggers the activation of a plant target of rapamicin complex that is expressed in primary meristems and integrates auxin and nutrient signalling by regulated protein translations [15]. Thus, nitrogen resources are pivotal for protein synthesis in the stem base of cuttings, wherein the predominant amino acids comprise glutamine (gln), glutamate (glu), asparagine (asn) and aspartate (asp) [8, 16]. Carbohydrate reserves and nitric oxide (NO) enhance resilience of plant tissues and survival of dark senescence [17–19]. As AR formation depends on protein re-synthesis [3, 4] from mobile or recycled nitrogen reserves such as asn [20, 21] these could be limiting in case of N deficiency and result in an accelerated leaf senescence [22, 23] differing from lateral roots formation, in this respect [24, 25]. So far nitrogen and carbohydrate limitations of AR formation have been shown in Pelargonium, Chrysanthemum, Poinsettia and Rosa [17, 26–28]. Enhanced AR formation at high nitrogen contents may be related to an increased basipetal transport of carbohydrates [26] and nitrogenous compounds [20] with limited knowledge of the causal mechanisms including transcriptome, hormone and metabolic adaptations. Using *Petunia hybrida* as a model plant three metabolic phases for AR formation were established [9] during which nitrogen supply was maintained at adequate levels. A dynamic depletion and replenishment of carbohydrates has been reported in course of dark exposure of the cuttings and their subsequent rooting under light with stimulating effect on root formation [29]. In addition, at adequate nitrogen levels a strong contribution of the polar auxin transport (PAT) to AR formation was shown by an early increase of indole-3-acetic acid (IAA) *in Petunia* [16]. Moreover, multiple transcriptome changes in auxin transport systems, auxin conjugation and auxin signal perception uncovered auxin as a key regulator of AR formation during sink establishment phase [9, 16, 30, 31]. At the sink side amino acids and nitrogen pools provide important N resources to meet the new demand for protein re-synthesis. In addition, variation in N resources may have an influence on auxin levels. It is supposed that prior to excision of cuttings various signalling hormones including cytokinin (CK) communicate the nitrogen availability from donor plant roots to axillary shoots [32] and that their activity can be related partially to glutamine metabolism [33]. CK’s are considered as auxin antagonists and important negative regulators of AR formation [34] that would counteract auxin distribution via down-regulation of PIN activity [35]. In contrast, CK’s are also considered as important signals for dedifferentiation processes during early induction of ARs [4] and are required for fine tuning of the auxin transport and biosynthesis during the formation of the quiescent centre in the adventitious root apex [36]. In this regard, shoot levels of both CK’s and gibberellins decline with an interrupted nitrogen supply to roots [37]. This complexity of functions of nitrogen metabolism interacting with plant hormone signalling might explain the lack of information on the influence of nitrogen nutrition of donor plants and dark exposure of cuttings on their nitrogen metabolism and AR formation. Therefore, the present study tested the hypothesis that enhanced N_t_ contents and dark exposure of cuttings influence their internal N-pools including free amino acids and affect early events of AR formation and further root development in *Petunia hybrida.*

## Results

### Anatomy of early events during AR formation at different nitrogen contents

**Fig. 1:**
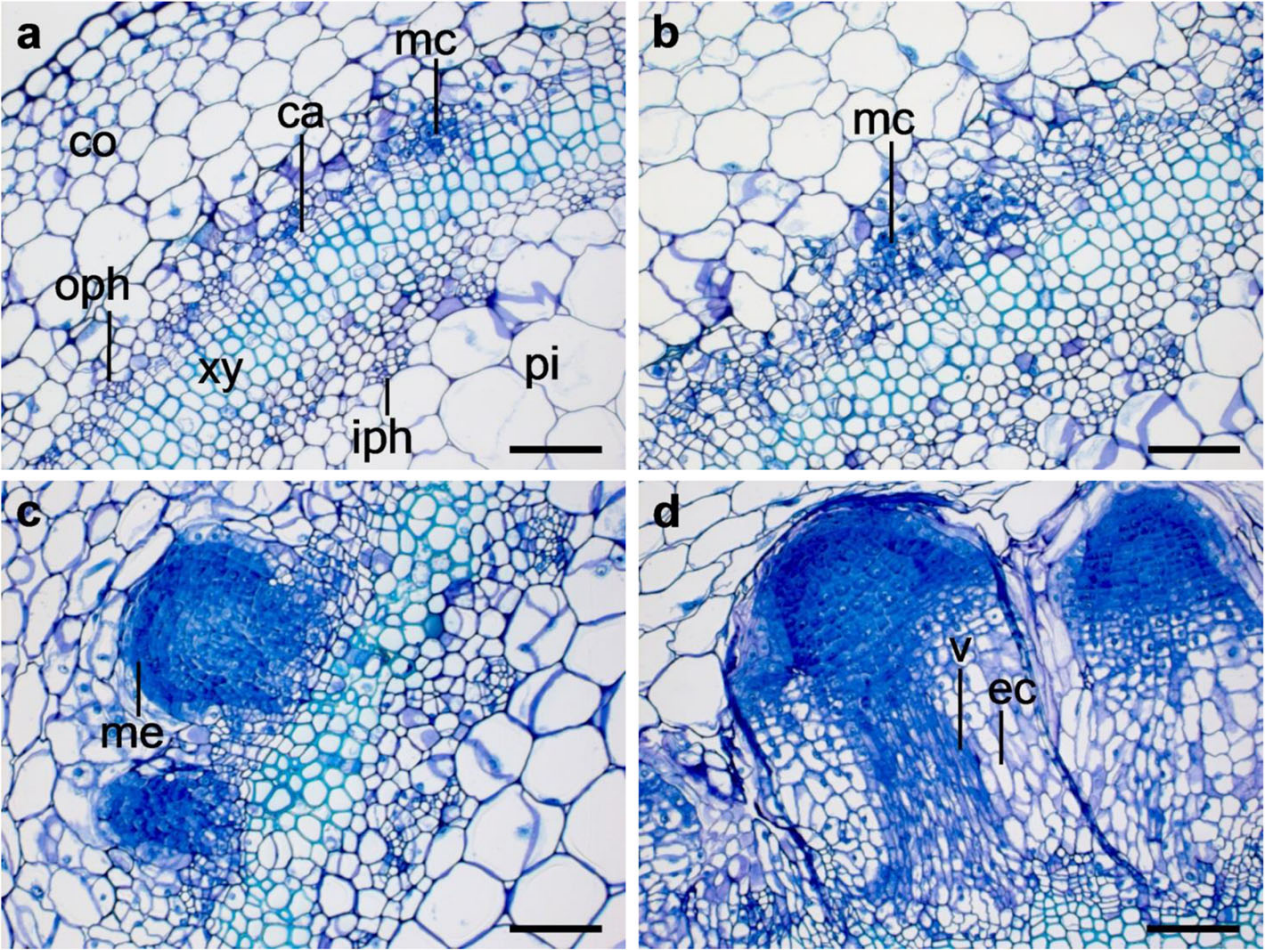
Influence of reduced total nitrogen on early cytological events of AR formation in *Petunia hybrida*. Cuttings with two nitrogen levels, (Panels **a**, **c**) N-low at 2570 μmol N_t_ and (Panels **b**, **d**) N-high at 3625 μmol N_t_ were excised from donor plants and immediately inserted into perlite for AR formation with assimilatory light. All micrographs represent cross-sections of the stem base from 1–4 mm above the excision site with the most advanced structures at (Panels **a**, **b**) 72 h post excision (hpe) and post insertion (hpin) and (Panels **c**, **d**) 168 hpe and hpin. Sections at 72 hpin (Panels **a**, **b**) show the typical stem anatomy with the cortex (co), the outer phloem (oph), the cambium (ca), the xylem (xy), the inner phloem (iph) and the pith parenchyma (pi) and first meristematic cells (mc) of developing root meristems, that is, small cells with a dense cytoplasm and a large nucleus. There are only slight differences between the nitrogen levels. Sections at 168 hpin reveal that with low N absorption (Panel **c**) first root meristems (me) appear, whereas with high N absorption (Panel **d**) first roots with vascular bundles (v) in the center surrounded by elongated cells (ec) of the elongation zone are visible. Bars represent 100 μm (Panels **a** to **d**). Remark: Ahkami et al. [8] show that at the time of excision (0 hpe) no meristematic cells of developing root meristems are present. Further details are presented in methods and with Additional file 1) Experiments of nitrogen preconditioning of cuttings and Additional file 2) Explanation of experimental designs for *Exp. 7: AR-N + CYT*, respectively

The histological examinations revealed that first meristematic cells of developing root meristems, i.e. small cells with a dense cytoplasm and a large nucleus were visible at 72 hpin in stem base sections of cuttings with two different total nitrogen (N_t_) contents (N_t_-low: 2570 μmol N_t_ g^−1^ DM, N_t_-high: 3625 μmol N_t_ g^−1^ DM) (Fig. 1a, b). At this time the difference between the nitrogen contents was marginal but at 168 hpin there was a significant difference between the two N_t_ levels. Whereas in the cuttings with the low N_t_ level only meristems were formed as the most advanced structures (Fig. 1c), the treatment with the high N_t_ level led to root formation with first cells characteristic for the elongation zone (Fig. 1d).

### Nitrogen pools in response to total N-absorption by cuttings

**Fig. 2:**
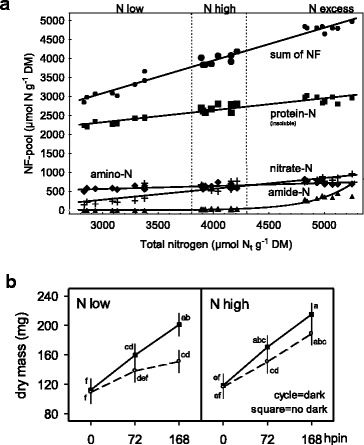
(Panel **a**) Relationships between accumulation of total nitrogen (N_t_) and N allocation to NF-pools. Axillary shoot tips of *Petunia hybrida* at excision time, N_t_ allocation to metabolic NF-pools classifying amide-N, amino-N, insoluble protein-N and the sum of NF-pools in response on three levels of N_d_ fertilization to donor plants (N low, N high, N excess). Linear correlation coefficients for nitrate-N (*r =* 0.91), amide-N (*r =* 0.88), amino-N (*r =* 0.74), insoluble protein-N (*r =* 0.96) and sum of NF-pools (*r =* 0.99) all *n* = 24, *p* < 0.05. (Panel **b**) Increase in shoot dry mass per cutting during AR formation (hpin) in response to two levels of N_d_ fertilization to donor plants (N low, N high) and to a pre-rooting dark exposure of cuttings (dark exposure - open circled symbols, no dark - squared symbols). Vertical bars represent 95 % confidence intervals of mean values and different lower-case letters indicate significant differences. Further details of experiments, regression equations and statistics are presented in methods and with Additional file 1) Experiments of nitrogen preconditioning of cuttings, Additional file 2) Explanation of experimental designs for statistical analyses and Additional file 3) Supplemental data of figure 2 for Panel **a**: *Exp. 1: NF-N*, and Panel **b**: *Exp. 9: NF-NDCR*, respectively

To characterise ranges of N_t_ contents and fractionated pools of nitrogen (NF-pools) within excised cuttings, their growth (number and biomass) with distinct N dosage (N_d_) regimes to donor plants was monitored (N_d_-low, N_d_-high, N_d_-excess: 55/90/179 mg N plant^−1^ week^−1^) (Fig. 2a). The N_t_ content of whole cuttings was determined on a dry mass (DM) basis (two sample sets, four biological replicates, *n* = 24). The N_d_-low, N_d_-high and N_d_-excess regimes produced N_t_ contents of cuttings of 3112 ± 187, 4034 ± 107, 5004 ± 119 μmol N_t_ g^−1^ DM, respectively. Considering all 24 samples, N_t_ changed between 2800 and 5300 μmol g^−1^ DM (Fig. 2a). Allocation of N_t_ to four NF-pools such as amide-N, amino-N, nitrate-N and insoluble protein-N was positively correlated with N_t_, as shown by linear regressions fitted between N_t_ and each NF-pool except amide-N. The amide-N remained very low both for N_d_-low (<1 μmol g^−1^ DM) and N_d_-high (27 ± 11 μmol g^−1^ DM) fertilization levels but rose steeply about 12-fold with excessive N-supply (340 ± 61 μmol g^−1^ DM). Nitrate-N ranging from 140 to 960 μmol g^−1^ DM was the most continuously increasing NF-pool (~7-fold increase) followed by protein-N as most abundant NF-pool while amino-N was most stable in the lowest range (∆N = 500–800 = 300 μmol g^−1^ DM).

### Shoot growth during AR formation

Shoot dry mass accumulation of excised cuttings was analysed during AR formation under diurnal light at three time points of insertion in perlite (0, 72, 168 hpin = hours post insertion) (Fig. 2b). In advance, cuttings of low and high N_t_ contents (N_t_-low, N_t_-high: 2900/3500 μmol N_t_ g^−1^ DM) were excised from donor plants grown with two fertigation rates (N_d_-low and N_d_-high: 46/78 mg N plant^−1^ week^−1^). Further, cuttings were exposed to dark (168 hpe = hours post exision). Both, cuttings without and with dark exposure did not differ in their initial dry mass at 0 hpin whereas a high N_t_ content enhanced dry mass by +10 % as a significant main N-effect. Dry mass accelerated mostly with increasing time of insertion up to 168 hpin by +79 %. Upon dark exposure this increase was reduced to +50 % while the delay for low N cuttings was most obvious at 168 hpin.

### AR formation with reduced N absorption and dark exposition of cuttings

**Fig. 3:**
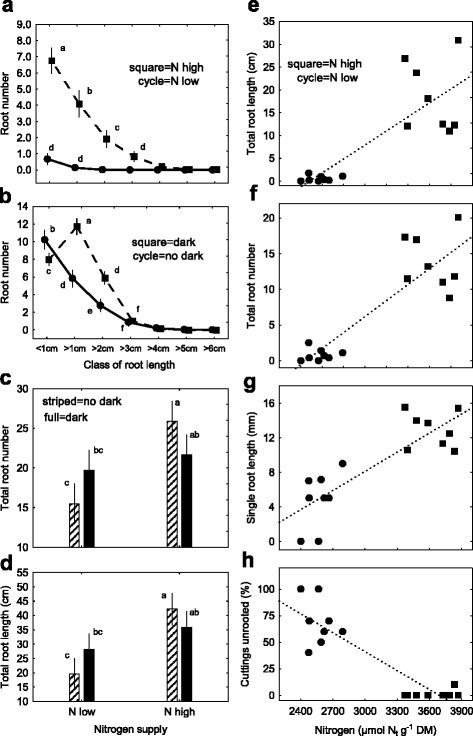
Adventitious root formation 384 hpe with cuttings of *Petunia hybrida*: (Panel **a**) Root number per length class (RNC) with two levels of total nitrogen content (N_t_) (cycles = N-low, squares = N-high). (Panel **b**) RNC response to dark exposure (squares = dark, cycles = no dark). (Panels **c** and **d**) Response of total root number (TRN) and total root length (TRL) on combinations of two N_t_ levels and dark exposure (striped bars = no dark, full bars = dark). (Panels **e** to **h**) Regression relationships between N_t_ contents in unrooted cuttings and their final root development at different N_d_ fertilization levels to donor plants (cycles = N-low, squares = N-high) (E: Total root length - TRL, F: Total root number - TRN, G: Mean length per single root – SRL, H: Unrooted cuttings – URC. (Panels **a** to **c**) Vertical bars represent 95 % confidence intervals of mean values and different lower-case letters indicate significant differences. (Panels **e** to **h**) Linear correlation coefficients: Panel **e** (*r =* 0.82), Panel **f** (*r =* 0.88), Panel **g** (*r =* 0.84) and Panel **h** (*r =* 0.89) all with *n* = 16, *p* < 0.05. Further details of experiments, rating of rooting (range per each class of root length = 1 cm), statistics and regression equations are presented in methods and with Additional file 1) Experiments of nitrogen preconditioning of cuttings, Additional file 2) Explanation of experimental designs for statistical analyses and Additional file 3) Supplemental data of figure 3 for Panel **a**: *Exp. 7: AR-N + CYT*; Panel **b** *Exp. 4: AR-D*; Panels **c** and **d**: *Exp. 2: AR-ND*, and Panels **e**, **f**, **g**, **h**: *Exp. 7: AR-N + CYT,* respectively

The effect of distinct N dosage to donor plants (N_d_-low, N_d_-high: 51/106 mg N plant^−1^ week^−1^) on N_t_ contents in cuttings (N_t_-low, N_t_-high: 2575/3629 μmol N_t_ g^−1^ DM) and AR formation was analysed. Total root number (TRN) developed per cutting at day 16 (384 hpe = hours post excision) differed significantly and reached 0.83 and 13.84 for low and high N_t_ contents, respectively (Fig. 3a). For detailed analyses, all roots of a cutting (i.e. TRN) were assigned to seven root length classes to determine the root number per length class (RNC). An increased root development for high N_t_ contents became evident in the length classes below 3 cm (TRN = ∑ RNC = 13.8 = 6.8 + 4.1 + 1.9 + 0.8 + 0,2 + 0 + 0), and a significant reduction was observed with low N_t_ contents (TRN = ∑ RNC = 0.83 = 0.68 + 0.14 + 0.01 + 0 + 0 + 0 + 0). Subsequently, the effect of a pre-rooting dark exposure of cuttings on AR formation was evaluated with cuttings grown at high N_d_ supply to donor plants to prevent N-limitation in cuttings (Fig. 3b). Excised cuttings were exposed to dark for 7 days (168 hpe) before perlite insertion. Sixteen days after excision (384 hpe) cuttings developed a TRN of 26.8 roots per cutting. At the same time (384 hpe) they developed considerably less roots (TRN = 19.9) when no dark was applied and perlite insertion was performed immediately after excision. The RNC analysis showed a prerequisite for pre-rooting dark treatment to increase root numbers in the second and third classes. However, cuttings without dark exposure developed in average two roots more in the first class <1 cm (Fig. 3b). These results substantiated by a larger experiment showed that an enhanced AR formation 384 hpe led to an increase of TRN by 73 % (15 vs. 26) and of TRL by 110 % (that is 20 vs. 42 cm total root length) with low and high N supply (N_d_-low, N_d_-high: 55/90 mg N plant^−1^ week^−1^), respectively (Fig. 3c, d). In contrast, in the same experiment a treatment combination of both the donor plant N dosages and dark exposure resulted in an increase of TRN by +30 % and of TRL by +40 % in cuttings with a low N_t_ while cuttings with a high N_t_ had slightly fewer (−14 %) and shorter roots (−15 %) (Fig. 3c, d). N_t_ contents ranged from 2400 to 3900 μmol N_t_ g^−1^ DM with N_d_-low and N_d_-high dosages (51/106 mg N plant^−1^ week^−1^) and no dark exposure, respectively (Fig. 3e, f, g, h). Regression analysis resulted in positive relationships for N_t_ with TRL, TRN and single root length (SRL) (Fig. 3e, f, g) and negative with percentage of unrooted cuttings at 384 hpe (Fig. 3h).

### Nitrogen content and N allocation in unrooted cuttings and during AR formation

**Fig. 4:**
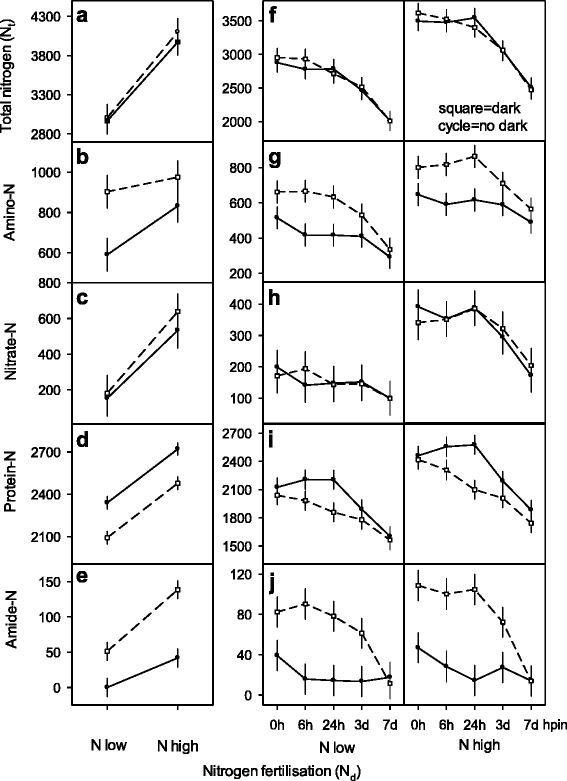
Change of total nitrogen (N_t_) and NF-pools in cuttings of *Petunia hybrida* with graduated nitrogen fertilisation (N_d_) to donor plants, dark exposure and AR formation. (Panels **a**, **b**, **c**, **d**, **e**): Full cycles and open squared symbols connected with full and intermittent lines show cuttings directly at severance (0 hpe) and after 168 h dark exposure (168 hpe), respectively. (Panels **f**, **g**, **h**, **i**, **j**): Full cycles and open squared symbols connected with full and intermittent lines represent cuttings at specific times post insertion for AR formation (0, 6, 24, 72, 168 hpin, corresponding days are shown in the x-axis: 3d, 7d) at 0 hpe and after 168 h dark exposure (168 hpe), respectively. All data are given in μmol N g^−1^ DM. Multifactorial ANOVA’s revealed significant main and interaction effects for N_d_-levels *p* < 0.01 (Panels **a**, **c**, **d**, **f**, **g**, **i**, **j**), for dark exposure *p* < 0.00001 (Panel **d**), for time post insertion *p* < 0.0001 (Panel **f**), for N_d_-levels × dark exposure *p* < 0.05 (Panel **b**, **e**), for N_d_-levels × time post insertion *p* = 0.01 (Panel **h**), for dark exposure × time post insertion *p* < 0.001 (Panel **g**, **i**, **j**). Vertical bars represent 95 % confidence intervals of mean values. Further details of experiments and statistics are presented in methods and with Additional file 1) Experiments of nitrogen preconditioning of cuttings, Additional file 2) Explanation of experimental designs for statistical analyses and Additional file 3) Supplemental data of figure 4 for *Exp. 6: NF-ND* (with Panels **a**, **b**, **c**, **d**, **e**) and for *Exp. 9: NF-NDCR* (with Panels **f**, **g**, **h**, **i**, **j**), respectively

The low and high nitrogen fertilisation regime (N_d_) of donor plants resulted in two different N_t_ absorption levels in cuttings while dark exposure of cuttings (as indicated by dotted lines in Fig. 4) remained without any impact on N_t_ contents (Fig. 4a, f). In contrast, after cuttings had been planted (inserted) into perlite and exposed to diurnal light a longer time post insertion resulted in continuous decreases of N_t_ contents in both immediately planted and dark pre-exposed cuttings (Fig. 4f). Allocations of N_t_ to single NF-pools were affected quite differently by external factors (i.e. N_d_ level, dark exposure). Amino-NF accumulated 12–25 % of N_t_, with both elevated N_d_ levels and dark exposure increasing amino-N in excised cuttings (0hpe) and in dark-exposed cuttings (168 hpe) (Fig. 4b, g). Amino-N decreased continuously with longer time post insertion (Fig. 4g). While 5–18 % of N_t_ were allocated to nitrate-N at the two N_d_ levels, the dark exposure of cuttings did not change nitrate-N content (Fig. 4c, h). In contrast, N_d_ level and increasing time post insertion resulted in distinct reduction of nitrate-N (Fig. 4h). Insoluble protein-NF was most abundant and comprised 50–70 % of N_t_ (Fig. 4d, i). An increased N supply enhanced protein-NF followed by a decrease upon dark exposure at both N_d_ supply levels (Fig. 4d). Dark treatment accelerated subsequent reduction of protein-NF with longer time post insertion and exposure to diurnal light. With both N_d_ levels, largest divergence occurred among cuttings with and without dark treatment 24 hpin. Finally, protein-NF similarly decreased 168 hpin to lowest amounts irrespective of dark treatment (Fig. 4i). Amide-N as smallest NF-pool accumulated merely 0–3.5 % of N_t_ (Fig. 4e, j). The N_d_ level and dark exposure raised the amide-NF up to 42 μmol in excised cuttings (0 hpe) and up to 140 μmol in dark-exposed cuttings (168 hpe). Dark exposure released much more amide-N within high N_t_ cuttings compared to low N_t_ cuttings (Fig. 4e). Regardless of a differential accumulation of amide-N in excised cuttings or after dark exposure, amide-N declined largely with increasing time post insertion (<18 μmol after 168 hpin) with both low and high N_d_ supply. Thereby different reduction rates proved dark exposure and time post insertion as variation sources (Fig. 4j).

### Change of free amino acids upon raising nitrogen nutrition and dark exposure

**Fig. 5:**
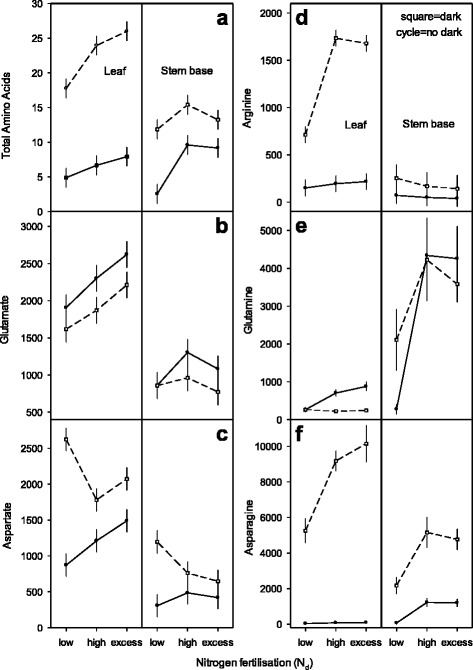
Change of proteinogenic amino acids in leaf and stem base with cuttings of *Petunia hybrida* in response to graduated nitrogen fertilisation (N_d_) to donor plants and dark exposure. Levels: low, high and excess N_d_ and 168 h dark exposure of cuttings (168 hpe, 10 °C). Panels **a** to **f**: Full cycles and open squared symbols connected with full and intermittent lines represent cuttings directly at severance (0 hpe) and after dark exposure, respectively. Total amino acids are given as μmol g^−1^ FM and single amino acids as nmol g^−1^ FM, respectively. Multifactorial ANOVA’s revealed significant main and interaction effects for N_d_-levels × dark exposure × tissue type *p* < 0.00001 to *p* < 0.05 (Panels **a**, **c**, **d**, **e**, **f**), for N_d_-levels × tissue type *p* < 0.00001 (Panel **b**), for dark exposure *p* < 0.000001 (Panel **b**). Vertical bars represent 95 % confidence intervals of mean values. Further details are presented in methods and with Additional file 1) Experiments of nitrogen preconditioning of cuttings, Additional file 2) Explanation of experimental designs for statistical analyses and Additional file 3) Supplemental data of figure 5 for *Exp. 3: AA-ND,* respectively

Free amino acids were examined in source and sink tissues (leaf and basal stem) applying three N_d_ levels to donor plants and dark exposure of cuttings (Fig. 5). Amino acids represent a pivotal part of the amino-NF and include shared amounts of total nitrogen in plant tissues ranging from 0.075 to 0.1075 % of N_t_. The levels of total and single amino acids glu, gln, asp, asn and arg responded both to graduated N_d_ fertilization of donor plants and dark exposure of cuttings (Fig. 5: squared symbols with dotted lines show dark responses). Further, specific patterns for amino acids were observed in the leaf and stem base of excised cuttings at 0 hpe and after dark exposure at 168 hpe, respectively (Fig. 5a to f). Hence, free amino acid (AA) considerably changed between 3 and 26 μmol g^−1^ FM and accumulated between 330 and 4260 nmol N g^−1^ FM = AA-N_t_. AA raised in excised cuttings (0 hpe) with enhanced N_d_ levels from 5 to 8 μmol g^−1^ FM in the leaf (equivalent to 590–1002 nmol N = AA-N_t_) and from 3 to 10 μmol g^−1^ FM in stem base (equivalent to 330–1550 nmol N = AA-N_t_). Dark exposure of cuttings during 168 h led to a general increase, up to 4-fold of AA released in leaf and stem base while a maximum of 26 μmol g^−1^ FM was observed in leaf at the highest N_d_ level (Fig. 5a). As primary metabolite in N assimilation, glutamate concentration was at higher levels in leaf compared to stem tissue and increased with the increasing N_d_ supply up to 2622 nmol glu g^−1^ FM. Further, elevated N_d_ levels raised glutamate in leaf of both excised (0 hpe) and dark exposed cuttings (168 hpe). While N supply increased from high N_d_ to excess N_d_, the glu reduction in stem base showed an interacting effect among N_d_-level and tissue type. Besides this, dark exposure decreased glu in leaf partly at distinct N_d_ levels (~380 nmol glu g^−1^ FM) (Fig. 5b). Altogether, glutamate accumulated between 73 and 250 nmol N_t_ in response to different nitrogen levels and dark exposure. In excised cuttings (0 hpe) aspartate also increased with increasing N_d_ supply. In contrast to glu, in leaf and stem tissue an increase was observed for asp in response to dark exposure. Highest asp-increase induced by dark was monitored with low N cuttings (Fig. 5c). Driven by three variation sources, asp accumulated between 32 and 276 nmol N_t_. Arginine (arg) in stem and leaf tissues of excised cuttings (0 hpe) accumulated at very low levels (<220 nmol arg g^−1^ FM), although elevated N_d_ levels increased arg slightly in the leaf and decreased it marginally in the stem. However, a dark exposure of cuttings (168 hpe) resulted in tremendous, almost 9-fold increase of up to 1734 nmol arg especially in leaf tissues grown at high N_d_ supply (Fig. 5d) while N amounts allocated to the arg pool changed between 10 and 461 nmol N_t_. Glutamine (gln) in cuttings (0 hpe) reflected the N_d_ levels to donor plants in a tissue specific manner. At elevated N_d_ level it increased in the stem about 17-fold up to 4341 nmol gln g^−1^ FM. The dark exposure (168 hpe) reduced gln in leaf tissue particularly at raised N_d_ levels, but caused contrasting responses in stem base tissue among low and raised N_d_ levels (Fig. 5e). The N_d_ level remained the critical source of variation for gln in stem base while dark exposure was the important factor in the leaf. Coherent N_t_ allocations to the glutamine pool ranged from 42 to 168 and 53 to 832 nmol N_t_ in leaf and stem tissue, correspondingly. Biosynthesis of asparagine involves aspartate and glutamine as the ammonium acceptor and donor, respectively. Asparagine (asn) increased in excised cuttings (0 hpe) with N_d_ levels in a tissue specific response. It remained very low in the leaf (≤93 nmol asn g^−1^ FM) and accumulated in significant amounts only in stem base at high N_d_ levels (≤1226 nmol asn g^−1^ FM). In contrast, a dark exposure (168 hpe = hde) caused strong specific increases in leaf and stem tissues at raising N_d_ levels with up to 10,147 nmol asn g^−1^ FM (Fig. 5f). Highest relative asn increase was established within the low N_d_ level especially in leaf and, to a lower extent in stem (leaf vs. stem: N_low_ = 164- vs. 33-fold, N_high_ = 121- vs. 4-fold; N_excess_ = 105- vs. 4-fold). Allocation of N_t_ to the asparagine pool reached a tremendous range among 7 and 2151 nmol N_t_. In addition, the impact of dark exposure on the amino acid accumulation coincided with nitrogen fluxes to the asparagine, aspartate and arginine pools, which hold up to 77 % of AA-N_t_ in the leaf, while stem tissue contained up to 74 % of the AA-N_t_ as glutamine and asparagine.

### Change of free amino acids in three different conditions

**Fig. 6:**
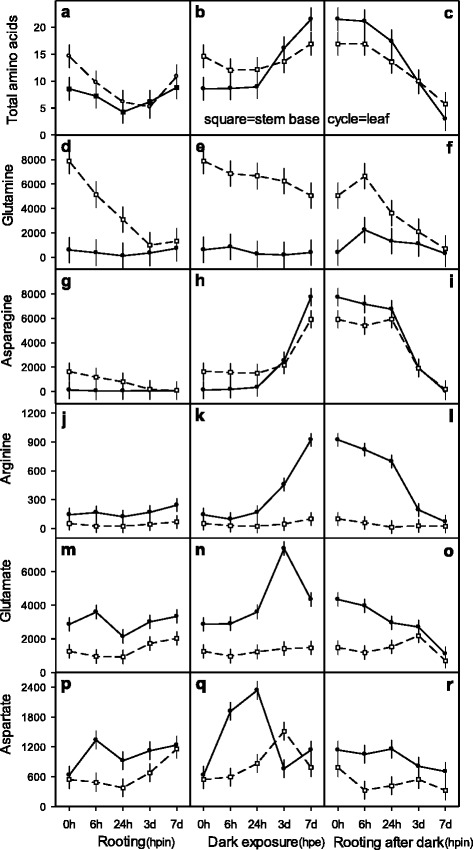
Course of total and single amino acids in leaf and stem base of *Petunia hybrida* cuttings exposed to three environment conditions **–** immediately planted for rooting, under dark exposure, planted for rooting after dark – and sampled at five exposition times (0, 6, 24, 72, 168 hpin or hpe, corresponding days are shown in the x-axis: 3d, 7d), respectively. Panels **a** to **r**: Full cycles and open squared symbols connected with full and intermittent lines represent leaf and steam base tissues, respectively. Total (free) amino acids are given as μmol g^−1^ FM and single amino acids as nmol g^−1^ FM, respectively. Multifactorial ANOVA’s revealed significant interaction effects for environment condition × exposition time × tissue type *p* < 0.00001 to *p* < 0.05 (Panels **a**-**b**-**c**, **d**-**e**-**f**, **g**-**h**-**i**, **j**-**k**-**l**, **m**-**n**-**o**, **p**-**q**-**r**). Vertical bars represent 95 % confidence intervals of mean values. Further details of experiments and statistics are presented in methods and with Additional file 1) Experiments of nitrogen preconditioning of cuttings, Additional file 2) Explanation of experimental designs for statistical analyses and Additional file 3) Supplemental data of figure 6 for *Exp. 5: AA-DCR*, respectively

To investigate the pattern of amino acid changes in leaf and basal stem of cuttings upon dark exposure a factorial design involving three environment treatments of cuttings (rooting, dark exposure, rooting after dark exposure) and five time points within each environment was studied in more detail (Fig. 6). With adequate N_d_ supply excised cuttings (0 hpe) initially contained 15 and 9 μmol AA g^−1^ FM in stem and leaf tissues, respectively (Fig. 6a). The immediate perlite insertion for rooting under diurnal light resulted at 24 hpe in significant AA reduction followed at 72 hpe by a significant recovery in leaf and stem (Fig. 6a). Under dark exposure, the AA levels initially declined slightly in stem and remained unchanged in leaf until 24 hpe. Prolonged dark exposure raised AA strongly up to 168 hpe in stem and leaf (+5 and +12 μmol), respectively (Fig. 6b). When dark pre-exposed cuttings were planted (i.e. inserted to perlite) and exposed to diurnal light this was followed by a 3- and 7-fold AA reduction in stem and leaf tissues, respectively (Fig. 6c). Altogether, increase and decrease rates of AA in leaf exceeded those in stem tissue (Fig. 6b, c).

At time of cutting excision, among free amino acids, *glutamine* was initially (0 hpe) highest in the stem base (stem/leaf: 7881/601 nmol gln g^−1^ FM). The immediate insertion for rooting initiated strong gln reduction to lowest levels in leaf (24 hpe) and stem base (72 hpe). Until 168 hpe gln recovered in leaf to initial levels but only slightly in stem (Fig. 6d). The dark exposure of cuttings (0 hde) reduced gln concentration in stem base by 36 % until 168 hde and decreased it to low initial levels in the leaf (Fig. 6e). The rooting after dark caused transient gln increases in stem and leaf followed by a reduction in both tissues to lowest levels at 168 hpe (Fig. 6f).

*Asparagine* was low in stem base and leaf tissue (0 hpe stem/leaf: 1634/122 nmol asn g^−1^ FM). A direct insertion for rooting caused asn reduction in stem and remained at low levels in leaf (Fig. 6g). During the first day of dark exposure asn decreased slightly in stem base and increased moderately in the leaf. In contrast, further dark exposure (168 hde) resulted in an increase of asn up to 23-fold in leaf, starting before 72 hde and 4-fold in stem starting after 72 hde (Fig. 6h). An insertion of dark exposed cuttings led to no change of asn in stem base up to 24 hpin and to a slight decrease in leaf (Fig. 6i) and strongly decreased, thereafter, both in stem (34-fold) and leaf (169-fold) and reached lowest levels at 168 hpin (Fig. 6i). *Arginine* was low (stem/leaf: 52/143 nmol arg g^−1^ FM) in stem base and leaf tissue and remained unchanged until 168 hpe (Fig. 6j). Dark exposure did not change arg significantly in stem while it increased strongly up to 9-fold until 7d (923 nmol arg) (Fig. 6k). With subsequent rooting after dark period arg reduction was evident in both stem and leaf during the first day. In the further course, arg remained at low levels in stem while a 10-fold reduction of arg occurred in leaf tissue (Fig. 6l).

*Glutamate* accumulated in stem at lower levels when compared to leaf (0 hpe stem/leaf: 1254/2861 nmol glu g^−1^ FM) (Fig. 6m). A direct insertion for rooting resulted in a slight increase of glu only in leaf and was reduced in both tissues to a transient minimum at day one, and restored finally beyond initial levels. Dark exposure resulted in transient increase of glu at 72 hpe in leaf (7378 nmol glu) while it remained unchanged in stem (Fig. 6n). With rooting after dark treatment, continuous glu reduction was observed in the leaf whereas, in stem glu declined only between 72 and 168 hpe (Fig. 6o).

*Aspartate* started at low levels (0 hpe stem/leaf: 542/629 nmol asp g^−1^ FM). With direct rooting asp elevated only in leaf (6 hpe: 1338 nmol asp), decreased 24 hpe transiently in stem and leaf, and increased 168 hpe beyond initial levels (Fig. 6p). At dark exposure, asp did not change at 6 hde in the stem, rose at 72 hde to a temporary peak and decreased finally to initial levels. In the leaf, asp increased immediately until 24 hde to a maximum (2336 nmol asp), decreased at 72 hde to a minimum (761 nmol asp) and ended at 168 hde in a 2-fold increase (1138 nmol asp) (Fig. 6q). During rooting after dark treatment, asp remained unchanged in the leaf at 24 hpe and resulted in a continual decrease afterwards. In stem base, a reduction of asp was followed by a peak at 72 hpe and decreased to lowest levels in all three environment conditions (Fig. 6r).

### Course of soluble protein during AR formation

**Fig. 7:**
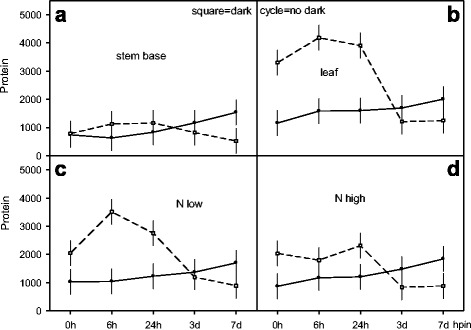
Course of soluble protein in leaf and stem base with cuttings of *Petunia hybrida*, grown at two N_d_ fertilisation levels (N low, N high) to donor plants and exposed to different environment conditions **–** immediately planted for rooting and planted for rooting after dark – and sampled at five exposition times (0, 6, 24, 72, 168 hpin, corresponding days are shown in the x-axis: 3d, 7d). (Panels **a** to **d**): Full cycles and open squared symbols connected with full and intermittent lines represent AR formation for 168 hpin directly after excision (0 hpe) and after dark exposure of cuttings (168 hpe, 10 °C), respectively. Multifactorial ANOVA’s revealed significant interaction effects for N_d_-level × dark exposure × exposition time *p* < 0.05 (Panels **c**-**d**), for dark exposure × exposition time x tissue type (Panels **a**-**b**). Vertical bars represent 95 % confidence intervals of mean values. Further details of experiments and statistics are presented in methods and with Additional file 1) Experiments of nitrogen preconditioning of cuttings, Additional file 2) Explanation of experimental designs for statistical analyses and Additional file 3) Supplemental data of figure 7 for *Exp. 8: PR-NDCR*, respectively

Soluble protein was examined in cuttings during AR formation under diurnal light. Environmental influence was assayed in a factorial design and included N_d_ supply levels to donor plants, dark exposure of cuttings, five time points after planting onto perlite for AR formation (i.e. insertion) and two tissues (Fig. 7). A direct insertion started at 0 hpe at 1.6-fold higher protein in leaf versus stem (1158/738 μg g^−1^ FM) (Fig. 7a, b). Then, its transient decrease in stem coincided at 6 hpin with an increase in leaf and rose at 168 hpin accordingly in both tissues to highest levels. In contrast, dark exposed cuttings (168 hde) at insertion (0 hpin) showed elevated protein in leaf only (3301 μg) followed by a decrease at 24 hpin (168 hde + 24 hpin = 192 hpe) and dropped to a level as in freshly excised cuttings (Fig. 7a, b). A direct insertion started 0 hpe at similar protein level for both N_d_ levels and increased continuously for 168 hpin (Fig. 7c, d). In dark exposed cuttings (168 hde), protein was increased at insertion (168 hde + 0 hpin) but did not differ for N_d_levels. However, at 6 hpin the low N_d_ supply resulted in a transient protein raise (3511 μg) while it remained unchanged at high N_d_ supply for 24 hpin. Thereafter, protein with both N_d_ levels dropped for 168 hpin to levels as in freshly excised cuttings (Fig. 7c, d).

## Discussion

### Early events of AR formation were delayed upon reduced N_t_ contents

Based on anatomical characteristics, the AR formation in petunia has been divided in the root initiation phase, the root primordium formation phase and the root elongation and/or emergence phase [8]. In the present investigation, two time points of AR formation have been chosen for the histological examination, the transition from the root initiation to the root primordium formation phase (72 hpin) and the root primordium formation to the root elongation phase (168 hpin). The results show that the course of AR formation at 72 hpin is similar to that reported by Ahkami et al. [8], irrespective of nitrogen treatment (Fig. 1a, b). At later stage (168 hpin) first roots were observed only in the treatment with high nitrogen level (Fig. 1d). Interestingly, this observation could not be confirmed with low nitrogen (Fig. 1c) demonstrating that a critical level of nitrogen is necessary to ensure proper root initiation and a subsequent differentiation into the complete body of the root. Delayed root formation at low nitrogen supply is most likely due to a regulation of root development and metabolic adaptations via nutrients and hormones [5, 8]. It has already been reported that an early transient rise in IAA and NO triggered anatomical changes for both AR and LR formation [11, 12, 38] and involved basipetal auxin transport before earliest cytological events of AR formation in petunia [16].

### Mobile nitrogen resources raised at elevated N_t_ contents

The ranges of total nitrogen accumulation in cuttings of axillary shoot tips of *Petunia hybrida* are comparable to published results for chrysanthemum, pelargonium and petunia [17, 26, 39]. An increased N_t_ absorption resulted in higher allocation towards soluble NF-pools while the allocation to insoluble protein-N decreased. However, along with increased N_t_ contents petunia cuttings accumulated more nitrate- and amide-N than amino-N (Fig. 2a). Planting of cuttings and subsequent exposure to diurnal light induced tremendous N_t_ decreases during initial rootless stages indicating that all NF-pools contributed to AR formation (Fig. 4f-j).

### Shoot growth during AR formation responds to N_t_ and dark exposure

A higher nitrogen content enhanced shoot dry mass accumulation by 10 % what is comparable to earlier reports [39]. Direct rooting (i.e. planting rsp. perlite insertion at 0 hpe) caused the most significant dry mass increase throughout until 168 hpin. In contrast, after a temporary dark experience (168 hpe) the subsequent shoot growth under diurnal light (168 hpin) was mostly reduced at a low initial N absorption of cuttings (Fig. 2b). Considering the simultaneous dark stimulation of AR formation with a low N absorption (Fig. 3c, d), this stays in accordance with the finding that dark exposure enhances the sink competitiveness of the rooting zone resulting in a higher proportion of dry mass allocation towards developing roots [40].

### Increased N absorption and dark treatment improves AR formation

The parameters of AR formation (TRN and RNC) were notably decreased at a lower N_d_ supply and reduced N_t_ absorption (Fig. 3a). This coincides with reports of a nitrogen limitation of AR formation in chrysanthemum, pelargonium, poinsettia and petunia [17, 26, 27, 39]. In the present study, AR formation in petunia was even reduced at low N_d_ supply (Fig. 3a) when N_t_ diminished just slightly below the recommended N_t_-range between 2750–5429 μmol N_t_ [39] (Fig. 3e, f, g, h). Then, cuttings revealed no phenotype above the critical level for deficiency symptoms of 1471 μmol N_t_ [41]. The dark stimulation of AR formation (Fig. 3b) supported earlier data of an increased, accelerated and synchronized rooting after dark exposure [29]. However, low and high initial N_t_ levels confirmed the high N effect on AR formation only as long as no dark exposure was applied (Fig. 3c, d).

### Nitrogen allocation mirrors N_t_ contents, dark exposure and period during AR formation

Nutrient concentrations, in particular nitrate, amino acids and N_t_ rapidly declined at early phases of AR formation indicating a high N demand [21, 42, 43]. As dark stimulation of AR formation involved a strong dark induced carbohydrate starvation [29] it further implied concurrent N fluxes obviously contributing to improved N availability in the rooting zone meeting the high N demand at early rootless phases [8]. Although darkness did not change N_t_ it reduced insoluble protein-N at both N_d_ supply levels (Fig. 4a, f, d, i) and warranted fluxes of released N into soluble amino-N and amide-N pools (Fig. 4b, g, e, j). This is in accordance with the reports that prolonged darkness affects proteolytic systems of cellular compartments and activates protein breakdown in response to carbohydrate starvation [44]. Thereby, total N and the nitrate-NF pool remains unaffected from darkness (Fig. 4a, f, c, h) since darkness prevents photosynthesis and concomitantly inactivates nitrate reductase and N assimilation [45].

### Amino acids show specific patterns for raised nitrogen nutrition and dark exposure

The amino acid levels in petunia increased with both enhanced N_d_ supply and dark exposure differing however, for both leaf and stem (Fig. 5a). Dark treatment was an exception among the other variation sources (N_d_-level × dark exposure × tissue type). Interestingly, leaf dark treatment led to highest amino acid concentration with excess N_d_ supply while amino acids in stem increased significantly, most of all with the low N_d_ supply. Recent reports showed that N_d_ supply is a pivotal external factor that initiates synthesis and raises amino acids along with internal nitrogen levels and altered gene transcription [46–48]. Amino acid levels also changed within different tissues and responded to other external factors as irradiance level, temperature shift and carbohydrate supply [49, 50]. Here, an increase of AA in leaf was dependent on the amount of nitrogen supplied with glu, asp and gln as major and arg as minor amino acids (Fig. 5b, c, d, e, f). In contrast, in stem an enhanced N_d_ supply resulted in high gln level (~17-fold) and low levels of glu and asn. These results are in agreement with previously reported data with gln as predominant amino acid followed by glu, asn and asp in the stem of petunia and of other plant species [8, 51]. A shift in the exogenous supply from high to excess N_d_ caused a stagnation (AA, gln, asn) or a slight decrease (glu, asp) of stem amino acids (Fig. 5a, b, c, e,f). This might be caused by an osmotic interference along with soil salt accumulation after excess N fertigation (Salt^KCl^ equivalent and growth of cuttings see Additional file 1). In this regard it was reported that the salt stres repressed long distance amino acid transport specifically in stem tissue [52].

Although dark exposure elevated AA in leaf and stem, single amino acids responded differently in each tissue. A general shift in N metabolism coincided with a strong carbohydrate starvation [29, 44]. The dark-induced increases of AA mainly comprised elevated asn, asp and arg levels with tissue specific responses at the different N_d_ supply levels (Fig. 5c, d, f). This is also strongly supported by reports that leaves senescing in the dark showed increases in asparagine and other amino acids [53]. Further, this is strengthened by data showing that in different plants asparagine synthetase genes (AS) are repressed by light and activated by dark. Moreover, AS genes are inversely correlated with carbohydrate levels in plants while exogenous sucrose repressed AS root gene expression [54–56]. Therefore, accumulation of free asparagine and arginine - having high nitrogen to carbon ratios - are assumed to serve as N storage compounds which buffer critical ammonium release in darkness. The ammonium release results from dark-induced proteolysis and amino acid deamination and is driven by accelerated respiratory carbohydrate demands for tissue survival during darkness [47, 57]. Interestingly, the dark-induced increase of asp was highest at a low N_d_ supply level in both leaf and stem tissues (Fig. 5c). This corresponds to an increase of asp found in response to low nitrogen stress among numerous metabolic adaptions in maize leaves [58]. However, while asn, asp and arg increased with dark exposure, the glu levels contrarily decreased in both tissues (Fig. 5b). Gln disappeared with dark exposure at high and excess N_d_ supply in leaf but remained unchanged in stem (Fig. 5e). In contrast, stem tissues of low N cuttings showed elevated gln in response to dark exposure. Taken together, under conditions of N deficiency (low N_d_ supply) the dark-induced increases of gln and asp were observed especially along with an AA rise in the stem (Fig. 5a, c, e). This might reflect an elevated dark-induced activity for N remobilisation by a temporary accumulation of metabolite precursors of asn synthesis like asp and gln [57]. In this regard, it was reported that protein degradation results in an increase in the Gln-synthetase/Glu-synthase (GS/GOGAT) cycle and that amounts of glu and gln increased, although overall amino acids decreased with N deficiency [59].

### Course of amino acids responded to distinct environment situations

Considering high and adequate N_d_ supply to donor plants, levels of AA and single amino acids displayed comparable ranges in two independent experiments (Figs. 5 and 6). Along with AA, gln and asn showed initial reductions in stem base up to 72 hpin (Fig. 6a, d, g), while glu and asp in stem base turned around to recover before 72 hpin (Fig. 6m, p). The amino acid changes in stem base are in agreement with the metabolic phases for sink establishment, recovery and maintenance suggested by Ahkami et al. [8]. With high N_d_ supply, the levels of amino acids changed differently in all tissues and conditions examined. Especially, at the end of dark exposure (168 hde/168hpe) tissue specific alterations of AA, gln, asn and arg were obvious (Figs. 5a, d, e and 6b, e, h, k). Furthermore, the respective courses of AA and single amino acids at earlier time points imply a switch in transcriptional regulation of amino acid metabolism (between 24hde and 72 hde) caused by the low energy status [60, 61] and with a dark induced carbohydrate depletion in leaf tissue [29, 44, 57]. Moreover, a subsequent basipetal allocation of amino acids (after 72 hde) might be induced with a preferred early sink establishment in the stem base region of root regeneration [40, 62]. Sink cell development strongly depends upon nitrogen which is transported mainly as gln or asn, and to lesser amounts as glu or asp via the phloem [21, 63]. However, during darkness N-rich amino acids (asn, arg) accumulated in the leaf (Fig. 6h, k) preventing ammonia toxification and using arg as precursor for a supposed NO signal cascade in early phases of AR formation [12, 13, 64]. The immediate and strong rise in asp level in leaf before 6 hde until 24 hde (Fig. 6q) and the subsequent increase in glu level from 24hde until 72hde (Fig. 6n) indicate a reprogramming of amino acid metabolism in darkness. In *Arabidopsis*, an induction of genes encoding enzymes of the asparagine biosynthetic pathway, such as glutamate dehydrogenase 2, aspartate aminotransferase, glutamate synthase and asparagine synthetase was found during 8d of dark exposure [60]. Further, it was reported that NAD(H)-dependent glutamate dehydrogenase is essential for plant survival during dark-induced carbon starvation [65]. Finally, a reciprocal regulation of asparagine synthetase genes by light and metabolites such as glutamine was shown [66]. This is in agreement with the decrease in courses of amino acid levels during AR formation under diurnal light and after the dark exposure (Fig. 6c, f, i, l and o) when carbohydrate biosynthesis was re-established [29].

### Soluble proteins undergo proteolysis in senescence

During cutting propagation developmental and abiotic stress signals include wounding, dark exposure and root induction with e.g. changing internal N resources. Thereby, reconvalescence and survival after senescence (developmental and/or dark induced) and AR formation depend on transcriptomic and metabolic adjustments to low energy status involving proteolysis and new protein synthesis [9, 67, 68]. In our study, soluble protein elevated during 7d (168 hpe = hpin) in both stem and leaf and at high and low N_d_ supply (Fig. 7). Suzuki and Kohno [21] suggested that nitrogen required by the growing parts (sinks) in the stem base comes from protein breakdown in source tissues (e.g. leaves). However, strong decreases of insoluble protein indicate N mobilisation during AR formation under diurnal light (Fig. 4i). At the two N levels proteolysis started 24 hpin and concurred with ~2-fold soluble protein rise (Fig. 7a, b, c, d).

In contrast, the pre-rooting dark exposure of cuttings likely involved an artificially, reversible senescence and transcriptome changes [67] that induced strong protein degradation [44] and changed amino acid metabolism under low energy stress [60, 68]. This can explain the high ~ 4-fold transient elevation of soluble protein especially in the leaf and at low N levels (Fig. 7b, c, d). Dark-induction of proteases in source tissues and corresponding sink establishment in the rooting zone with following enhanced flux of assimilates under subsequent light [29, 40, 62] could comprise degradation of chloroplast proteins like Rubisco [69] and involve senescence-associated vacuoles [70], that release peptides and amino acids for phloem upload and transport to new sinks in basal stem [9, 62]. This explains the positive correlation between proteins, amino acids as well as N_t_ and AR formation in petunia (Fig. 3e, f, g), minirose [28] and chrysanthemum [26]. While protein degradation contributes to auxin signalling by the ubiquitin-proteasome pathway [71] proteolyses selectively involve ubiquitin ligases (E3s) that control cell cycle by targeting cyclin-dependent kinase inhibitors [72] and thus influence lateral root and AR development [73, 74]. Hence dark stress might modulate auxin-mediated cell cycle progress and accelerate AR formation in petunia [29].

### Darkness induces the accumulation of free amino acids

**Fig. 8:**
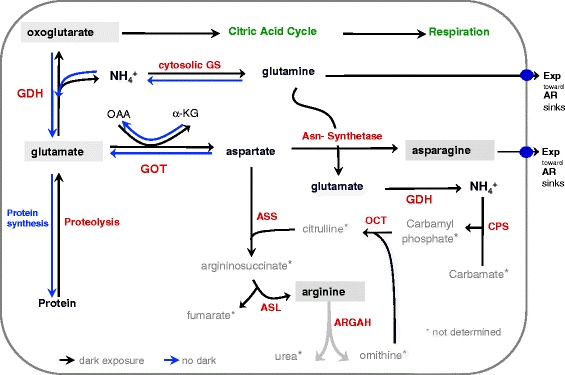
Schematic representation of metabolic processes of N-remobilisation during dark-induced proteolysis on carbohydrate depletion. Extended darkness results in carbohydrate depletion and low energy status that triggers proteolysis and amino acid catabolism with glutamate as primary amino acid. GDH deaminates glutamate to oxoglutarate and free NH4+ that is captured by cytosolic glutamine-synthetase (GS) to produce glutamine and glutamate. Glutamine and aspartate are used by asparagine synthetase to generate glutamate and asparagine. Both, asparagine and glutamine are exported to sink tissues as a nitrogen resource. Arginine accumulates in the urea cycle to prevent NH4+ accumulation to toxic levels. *Enzymes*: GDH – Glutamate-dehydrogenase, GS – Glutamine-synthetase, Asn – Asparagine-synthetase, GOT – Glutamic-oxaloacetic transaminase = AspAT – Aspartate-aminotransferase, ASS – Argininosuccinate-synthetase, ASL – Argininosuccinate-lyase, ARGAH – Arginine-amidohydrolase, OCT – Ornithin-carbamoyl-transferase, CPS – Carbamoylphosphat-synthetase; *Metabolites*: α-KG – α-Ketoglutarate, OAA – Oxaloacetic acid, CP – Carbamoylphosphate, CT – Carbamate; **●** – Transport protein in cell membrane, *black arrow* – *dark exposure*, *blue arrow* – *no dark*

Based on our findings, a model for dark induced accumulations of free amino acids is presented (Fig. 8). Extended darkness results in carbohydrate depletion by rapid glycolysis and low energy status triggering the proteolysis and amino acid catabolism with glutamate as primary amino acid. By the action of aspartate-aminotransferase (AspAT) = glutamic-oxaloacetic transaminase (GOT) the amino group (−NH2) of glutamate is transferred to oxaloacetate (OAA) producing aspartate and alpha-ketoglutaric acid (α-KG). This is supported by the accumulation of aspartate (6–24 hde) and glutamate (24–72 hde) (Fig. 6n, q). Further, NAD(H)-dependent glutamate dehydrogenase (GDH) deaminates glutamate to oxoglutarate and free NH4+ which in turn is captured by cytosolic glutamine-synthetase (GS) to produce glutamine and glutamate being exported to sink tissues. Glutamine and aspartate are used by asparagine synthetase to generate glutamate and asparagine (24-168hde) while asparagine is exported to sink tissue as a nitrogen resource for AR formation. This process contributes also to the transient accumulation of glutamate (24-72hde) (Fig. 6n) which is again catabolised by GDH to oxoglutarate and free NH4+. To prevent NH4+ accumulation to toxic levels, NH4+ and carbamate are bound by the action of carba-moylphosphat-synthetase (CPS) to carbamoylphosphate. Finally, arginine accumulated (24-168hde) (Figs. 5d and 6k) in the urea cycle and might contribute to endogenous NO synthesis which is essential for the IAA signal transduction in AR formation.

## Conclusions

In the present study, we first demonstrated that enhanced nitrogen concentrations of cuttings did not modify the establishment of new root meristematic cells until 72 h. However, subsequently, an enhanced N_t_ of cuttings accelerated further early anatomical events of differentiation during AR formation in the stem base until 168 h, which was followed by a strong rise in the number of emerged ARs and their total length at 384 h. Also, dark exposure stimulated AR formation in cuttings and this was most pronounced with a low N_t_. The results further clearly showed, that the graduated N_d_ supply to donor plants and a dark exposure of cuttings changed the accumulation of N_t_ and N allocations to metabolite pools like soluble protein, amino N and major amino acids of the primary N metabolism as glu, gln, asp, asn and arg in stem and leaf tissues, respectively. In this regard, developmental and reversible senescence adaptations of N metabolism with remobilization of nitrogen compounds are shown to facilitate AR formation in cuttings. Further analysis of metabolic responses and of the transcriptome including plant hormone related pathways should be combined with functional analysis of candidate factors to disentangle how nitrogen remobilization stimulates AR formation in cuttings. Finally, it was concluded that refined strategies for highest propagation rates should include nitrogen fertilization of donor plants and subsequent dark storage of cuttings. For an enhanced survival and rooting capacity improved quality assessment systems are required [75]. They can consider amino acid profiles and/or composite nitrogen pools as metabolic indicators of cutting quality along with carbohydrates in the global production chains for young plants.

## Methods

### Donor plant cultivation

Young plants of *Petunia hybrida* cv. Mitchell (Genotype W115) were grown from seeds in vitro and then transferred to the greenhouse [29]. Subsequently, they were transplanted into 1.1 l pots filled with a special mix of peat based substrate without slow release fertilizers (Einheitserde Typ ED-73 with Optifer, Patzer, Sinntal-Jossa, Germany). A continued growth of side shoots from axillary nodes was induced by weekly excisions of all mature shoot tips. Every excised shoot tip contained 4–5 leaves of similar size while two axillary nodes remained on its donor shoot providing sites for the regeneration of new buds and shoots. The greenhouse climate of the stock plants was regulated by shading from excess natural irradiance (photon flux density (PPFD) > 720 μmol m^−2^ s^−1^ (400 – 700 nm)), day length restriction (10 h day light regulated by an opaque curtain) and heating/ventilation temperature set points (day 20/22 °C and night 16/18 °C). The average daily light integrals (DLI), relative humidity and air temperatures in the greenhouse were registered for the 21 day periods while new side shoots developed at the donor plants before the date of excision. These 21 day averages ranged from 2.4 to 14.2 mol m^−2^ d^−1^ DLI (400–700 nm), from 55 to 77 % relative humidity and from 17 to 23 °C air temperature within the four cultivation periods (for details see supplemental data in Additional files 1, 2 and 3).

### Conditioning the physiology of donor plants and cuttings

All donor plants initially were cultivated starting with a uniform nutrient availability, pH and osmotic properties in the peat substrate (Einheitserde Typ ED-73 in mg l^−1^: 167 NO_3_-N, 75 P, 133 K, 4.1 pH, salt equivalent 1.83 g l^−1^ KCl). Fifteen donor plants were assembled in each of two or three treatment plots for graduated nitrogen fertigation regimes while the different treatment plots comprised each of four replication plots (3 N-treatments × 4 replications × 15 plants = 180 plants assembled in 12 plots or 2 N-treatments × 4 replications × 15 plants = 120 plants assembled in 8 plots). The excised leafy cuttings were either transplanted immediately into a perlite substrate for adventitious root (AR) formation or exposed to a dark cool environment (see details below) prior to transplanting for AR formation.

### Cultivation of donor plants at graduated nitrogen fertilization dosages

To differentiate nitrogen levels in excisable leafy cuttings, donor plants received up to three fertigation regimes which differed in nitrogen dosages only and provided a normal supply of all remaining nutrients at similar pH levels. In order to fine-tune nutrient dosages to environmental changes in biomass growth, peat substrate was controlled by regular chemical analyses according to Zerche and Druege [27].

The nitrogen dosages were differentiated about 14 days after transplanting and delivered by nutrient solutions (150 ml nutrient solution per plant and week) at three graduated N-dosage regimes: N_d_-low 41–64, N_d_-high 78–109 and N_d_-excess 150–179 mg N per plant and week, respectively. By this way specific total N dosages accumulated during the different periods of donor plant cultivation prior to each date of shoot tip excision (for details see Additional file 1). With the longest period of cultivation to the excision date each donor plant received totally 973, 1978 and 3611 mg N in the N_d_-low, N_d_-high and N_d_-excess dosage regimes, respectively.

The nutrient solutions for the different nitrogen regimes were prepared by combinations of suitable and balanced amounts of NH_4_NO_3_ (9 % NO_3_–N + 9 % NH_4_–N) and Ca(NO_3_)_2_ (19 % Ca + 14.5 % NO_3_–N + 1 % NH_4_–N) according to Zerche and Druege [27]. All other macro- and micronutrients were added at equal amounts to these nutrient solutions using the complex fertilizer Ferty Basis 1 (14 % P_2_O_5_, 38 % K_2_O, 5 % MgO, and micronutrients; Planta Düngemittel GmbH, Regenstauf, Germany).

### Excision of cuttings, dark exposure and illuminated perlite insertion for AR formation

The excision of mature cuttings (shoot tips with 4–5 leaves) started only after donor plants had received at least 4 h day light [29]. Cuttings were collected per nitrogen level and replication plot. For dark treatment they were placed in non-perforated plastic bags in cardboard boxes and immediately relocated to a cooled dark cabinet at 10 °C for 7 days. For AR formation under diurnal light cuttings were inserted in trays filled with perlite (Granule size 0–6 mm; Knauf Perlite GmbH, Dortmund, Germany) and placed in a climate chamber with diurnal light/dark cycles [29, 40]. Cuttings were both excised and directly inserted/planted in perlite for AR formation during 16 days or were kept in darkness for 7 days and then inserted for AR formation during 9 days (16 days from excision). At each excision date the defined number of cuttings was collected per replication plot and treatment combination pertaining to paralleled samplings for examinations of (i) histological stages (2 cuttings), (ii) rooting capacity (10 cuttings) and (iii) biomass growth (10 cuttings) or biochemical analyses of (iv) NF-pools (10–15 cuttings), (v) free amino acids (2 or 5 cuttings) and (vi) soluble proteins (2 cuttings). Thereby multifactorial experiments electively considered single treatments and or combinations of environmental factors as nitrogen level, time at dark exposure and or time after insertion (planting) and exposure to diurnal light for AR formation. Uniform time-scales were adapted to all samplings according to Klopotek et al. [29, 40] utilising either hours post excision (hpe), hours dark exposure (hde) during dark treatment and hours post insertion (hpin) during AR formation, respectively.

### Evaluating the capacity of AR formation

For adventitious root formation ten cuttings per replication plot were inserted into perlite trays, watered once a day and covered with clear hoods to conserve humidity. The cultivation for AR formation continued in a phytotron for 16 or 9 days (384 hpin or 216 hpin) as defined in individual experiments. The following growth conditions were maintained at diurnal cycles: 10 h light/14 h dark, temperature 22 °C day/20 °C night, humidity 85 % day/60 % night. Fluorescence tubes (Master TLD 58 W 830, warm-white, Phillips, The Netherlands) provided the light at 100 μmol m^−2^ s^−1^ PPFD (400–700 nm) [29, 40].

The capacity of AR formation was rated uniformly at 384 hpe (hours post excision) and according to standard procedures [27, 29]. This constant time period of 384 hpe coincided either with 384 hpin (hours post insertion into perlite) in case of direct planting for AR formation under diurnal light or included an initial time of 168 hde (hours dark exposure) which was finished with planting for AR formation under diurnal light for 216 hpin (i.e. 384 hpe = 168 hde + 216 hpin) as defined in the experiment. Accordingly the number of adventitious roots was counted at each inserted cutting while all the roots were assigned to their classes of root length in 10 mm ranges. The total number of roots per inserted cutting (TRN), the root number per length class per cutting (RNC), the mean single root length (SRL), the total root length per inserted cutting (TRL) and the percentage of unrooted cuttings (URC) were calculated as follows:

Total root number per inserted cutting (TRN):

$$ TRN={\displaystyle \sum {n}_i/10} $$

Root number per length class per cutting (RNC):

$$ RNC={\displaystyle \sum {\left({n}_{Lx}\right)}_i/10} $$

Single root length (SRL, mm):

$$ SRL={\displaystyle \sum {\left[{\displaystyle \sum \left({n}_{Lx}{L}_x\right)}\right]}_i/{\displaystyle \sum {n}_i}}*10 $$

Total root length per cutting (TRL, cm):

$$ TRL= TRNxSRL/10 $$

Percentage of unrooted cuttings (URC, %):

$$ URC={n}_Zx100/10 $$

where *n* stands for the number of roots of each inserted cutting, *i* is each of 10 cuttings inserted per replication plot, *L*_*x*_ is the mean length of the length interval (e.g. 1.5 cm for a length range of 1.0–2.0 cm), *n*_*Lx*_ is the number of roots in the specific length interval, and *n*_*z*_ is the number of unrooted cuttings out of 10 cuttings per replication.

### Histological examination of rooting zone

Basal stem tissue of cuttings (5 mm) was collected during AR formation at 72 hpin (=72 hpe) and 168 hpin (=168 hpe) from two nitrogen treatments (nitrogen absorption level N-low at 2570 μmol N_t_ and N-high at 3625 μmol N_t_). Sample preparation and histological analysis was essentially accomplished as described in detail by Haensch [76]. In brief, basal segments were embedded in hydroxyethylmethacrylate (Histo-Technique-Set Technovit 7100; Kulzer, Wehrheim, Germany), cut into 6 μm sections and stained with 0.05 % toluidine blue O (Serva, Heidelberg, Germany). Microscopic analysis was carried out using an AxioImager A1 microscope in combination with an AxioCam MRc5 camera (Carl Zeiss, Jena, Germany) and the software AxioVision Rel. 4.7.1. Original images were supplemented with embedded annotations and a scale bar.

#### Biochemical measurements and analyses

##### Total nitrogen and fractionated pools of nitrogen (entire shoot tip)

To analyse total nitrogen (N_t_), four fractionated pools of nitrogen (NF-pools: soluble: amide-N, nitrate-N, amino-N and insoluble protein-N), with 10–15 cuttings were sampled per replication plot and gently dried at 60 °C for 48 h. An adapted Kjeldahl method [77] was used to digest 300 mg of a dried and pulverised subsample (1 g Wieninger’s reagent (w/w 96,5 % Na_2_SO_4_; 1,5 % CuSO_4_; 2 % Se) + 2 g K_2_SO_4_ + 10 ml 98 % H_2_SO_4_) and to determine total nitrogen (N_t_) via water steam distillation (Büchi Kjeldahl Line: K-435, B-324, Titrino 719S; Büchi & Metrohm, Switzerland). Another subsample of 500 mg was extracted in 50 ml of 1 % w/v KAl(SO_4_)_2_ for 30 min (Shaker KL 2, Edmund Bühler GmbH, Germany) and passed through a folded filter (Schleicher&Schüll 595 ½). To determine the nitrogen in each NF-pool, extract aliquots of every 10 ml were used for the three distillations: (i) purely for amide-N, (ii) after nitrate reduction by Devarda’s alloy (w/w 45 % Al, 50 % Cu, 5 % Zn), and (iii) after digestion of amino-N as with N_t_. Finally, (iv) insoluble protein-N was analysed within the filter cake and the filter paper (Digestion: 2 g Wieninger’s reagent + 4 g K_2_SO_4_ + 20 ml 98 % H_2_SO_4_). The N-content of each NF-pool, the sum of NF-pools and the total nitrogen (N_t_) were calculated as μmol N g^−1^ dry mass (DM). The nitrogen recovery rate with the sum of NF-pools was accepted while the independently determined value for N_t_ coincided in a range of 100 ± 5 %.

##### Soluble protein (leaf and stem base)

For soluble protein analyses during AR formation, two cuttings were collected per replication plot at the defined time points. From the fully developed, second oldest leaf of both cuttings, every two discs were excised and combined as one leaf sample (2 × 2 discs, Ø 4 mm, 45 ± 12 mg FM), while left and right lamina tissues were sampled equidistantly from the mid-vein and leaf borders. The basal stem segments of the two cuttings were combined as one stem sample (2 × 5 mm, 114 ± 31 mg FM). All samples were frozen immediately in liquid nitrogen and stored at −80 °C, homogenised in a cryogenic ball mill (Retsch MM 301, Haan, Germany) and extracted in 1 ml buffer containing 50 mM Tris–HCl, pH 6.8, 5 mM MgCl_2_, 5 mM Mercaptoethanol, 15 % glycerine, 1 mM EDTA, 1 mM EGTA, 0.1 mM Pefabloc. Extract aliquots of 0.1 ml were mixed with 1 ml Bradford reagent (Sigma-Aldrich Chemie GmbH, Munich, Germany) and soluble protein [78] was quantified at 595 nm via spectrophotometer (Spekol 11, Carl Zeiss, Jena, Germany). Concentration of protein was calculated with an authentic standard (BSA - Albumin from bovine serum, Sigma-Aldrich, Germany) and expressed in μg g^−1^ FM.

##### Free proteinogenic amino acids (leaf and stem base)

For free amino acid analyses in leaf and stem base tissue, every five (*Exp. 3 AA-ND, details of design see ahead or in additional files*) or two (*Exp. 5 AA-DCR*) cuttings were collected per replication plot and at defined time points. Two leaf discs were excised (1 × 2 discs, at left and right sides of the mid-vein, Ø 4 mm, 25 ± 5 mg FM) with the fully developed, second oldest leaf of each cutting while five (*Exp. 3 AA-ND*) or two (*Exp. 3 AA-DCR*) replicated leaf samples were obtained. The basal stem segment of each cutting (1 × 5 mm, 50 ± 15 mg FM) contributed five or two replicated stem samples, respectively. Samples were frozen immediately in liquid nitrogen and stored at −80 °C. A homogenization of each sample in a cryogenic ball mill (Retsch MM 301, Haan, Germany) preceded the extraction with 500 μl 80 % ethanol in a thermostat shaker (80 °C, 45 min) (Thermomixer Comfort, Eppendorf, Germany). After 15 min cooling, samples were centrifuged for 5 min at 14,000 rpm and 4 °C. The supernatant was transferred into a new tube and evaporated in a vacuum concentrator (Savant SpeedVac, SPD111V, Thermo Fisher Scientific, Germany) at 50 °C to dryness (60–90 min). The dry residue was resolved in 150 μl (*Exp. 3 AA-ND*) or 250 μl (*Exp. 5 AA-DCR*) and either frozen at −20 °C until analysis or directly centrifuged for 1 min at 14,000 rpm. An aliquot was derivatised with 6-aminoquinolyl-N-hydroxysuccinimidyl carbamate (AQC) according to Ahkami et al. 2009 [8]. Individual contents of 18 amino acids and the nitrogen portion they reserved were calculated as nmol g^−1^ FM and as nmol N_t_ g^−1^ FM. Total free amino acids (AA) and their related total nitrogen contents (AA- N_t_) were specified in μmol g^−1^ FM and in nmol AA-N_t_ g^−1^ FM, respectively.

##### Statistical analyses

Nitrogen absorption (N_t_) and pools of nitrogen (NF-pools), adventitious root (AR) formation, early cytological events (CYT), free amino acids (AA) and soluble proteins (PR) were studied in response to distinct or combined environmental factors like donor plant nitrogen fertigation (N_d_), and time post excision of cuttings with exposure to dark (D) and post insertion during cultivation of cuttings at diurnal light (CR) totally in ten experiments. In these experiments factorial designs were applied which are specified in Additional files 1, 2 and 3 and assigned to results in Figs. 1, 2, 3, 4, 5, 6 and 7 (*Exp: 1 NF-N, 2 AR-ND, 3 AA-ND, 4 AR-D, 5 AA-DCR, 6 NF-ND, 7 AR-N ± CYT, 8 PR-NDCR, 9 NF-NDCR*). The analyses were accomplished by ANOVA/MANOVA in the data analysis software system STATISTICA [79]. Linear correlation and regression analyses between total nitrogen (N_t_) and N_t_ allocation to the NF-pools as well as between N_t_ and the rooting of the cuttings were performed in the Basic Statistics module. Differences among mean values were assessed with Tukey’s honestly significant difference (HSD) test (α = 0.05), and correlation coefficients were considered significant at P ≤ 0.05. In individual figures, vertical bars depict 95 % confidence intervals of least square mean (LSM) values as uniform standard measure of dispersion of a current interaction effect. Thereby calculations of 95 % confidence intervals of LSM values were accepted as default software mode. In appropriate cases calculations of 95 % confidence intervals of weighted mean values where conducted to show variably deviations in dispersion of single mean values in the current interaction effect. Different lowercase letters on data points denote significant differences at *P* ≤ 0.05.

## Additional files

Additional file 1: Experiments 1-9 of nitrogen preconditioning of cuttings. (PDF 22 kb)
Additional file 2: Explanation of experimental designs for statistical analyses. (PDF 13 kb)
Additional file 3: Supplemental data of Figures 2 to 7. (PDF 31 kb)

## Abbreviations

AA: Free amino acids
AR: Adventitious root
arg: Arginine
AS: Asparagine synthetase genes
asn: Asparagine
asp: Aspartate
CK: Cytokinin
CYT: Cytological events
GA: Gibberellins
gln: Glutamine
glu: Glutamate
hde: Hours of dark exposition
hpe: Hours post excision
hpin: Hours post insertion (i.e. after planting in perlite substrate for AR formation under diurnal light)
IAA: Indol-3-Acetic-Acid
N_d_ level: Nitrogen supply level to donor plants
NF-pools: Fractionated pools of nitrogen
NO: Nitric oxide
N_t_: Total nitrogen
PAT: Polar auxin transport
PIN: Auxin transport protein PIN-FORMED
PR: Soluble proteins
RNC: Root number per length class per cutting
SRL: Single root length
TRL: Total root length per inserted cutting
TRN: Total root number per cutting
