# Supplementary material for: Nitrogen remobilisation facilitates adventitious root formation on reversible dark-induced carbohydrate depletion in Petunia hybrida
Source: BMC Plant Biol. 2016 Oct 10;16:219. doi: 10.1186/s12870-016-0901-6 (PMC5056478; doi:10.1186/s12870-016-0901-6)
Supplement: Additional file 1: — Experiments 1-9 of nitrogen preconditioning of cuttings. (PDF 22 kb) [file 12870_2016_901_MOESM1_ESM.pdf]

Additional file 1: *Experiments of nitrogen preconditioning of cuttings via differential nitrogen fertigation of donor plants*

(*Growth of cuttings - i.e. axillar shoot tips - and Explanations of Experiment Codes see at next page)*

Experiment (Code)	Date of shoot tip excision	DLI* before excision	Humidity before excision*	Temp before excision*	Period of donor plant cultivation	Nitrogen supply mg N[z] plant^{-1} week^{-1}	Total N-dosage cumulated mg N plant^{-1}	Cutting Yield cumulated number plant^{-1}	Biomass Yield cumulated g plant^{-1}
	dd/mm/yy	av. 21d*	av. 21d	av. 21d	days (total)	low/high/excess**	low/high/excess**	low/high/excess	low/high/excess
1 (NF-N)	06+20/09/06	5.2 + 5.2	77 + 70	19 + 20	91 + 105[z]	55 / 90 / 179	768 /1253/2516	76 / 87 / 85	168 / 208 / 175
2 (AR-ND)									
3 (AA-ND)	22/11/06	2.4[a]	68	17	168	41 / 82 / 150	973/1978/3611	101 / 123 / 124	206 / 273 / 246
4 (AR-D)	12+19/06/07	13 +13.8	58 + 57	23 + 23	69 + 76[z]	0.10-0.30% NL-Hakaphos	n.d. no data	81 + 94	242 + 274
5 (AA-DCR)									
6 (NF-ND)	10/07/07	13.0	55	22	49	64 / 109 / n.e.	450 / 760 / n.e.	59 / 68 / n.e.	128 / 164 / n.e.
7 (AR-N+CYT)	14+21/08/07	10.6+11.6	58 + 57	22 + 22	84 + 91[z]	51 / 106 / n.e.	660 / 1381/ n.e.	115 / 146 / n.e.	206 / 327 / n.e.
8 (PR-NDCR)	09/10/07	7.2	61	19	140	44 / 105 / n.e.	886 / 2091/ n.e.	187 / 248 / n.e.	289 / 510 / n.e.
9 (NF-NDCR)	10/06/08	14.2	57	23	63	46 / 78 / n.e.	417 / 702 / n.e.	36[b] / 45[b] / n.e.	60[b] / 96[b] / n.e.

Experiment (Code)	Salt äq. KCl g l^{-1} peat, *donor plant substrate*	current NO_3-N mg l^{-1} peat	current P mg l^{-1} peat	current K mg l^{-1} peat	current pH $CaCl_2$
	low/high/excess**	low/high/excess**	low/high/excess**	low/high/excess**	low/high/excess**
1 (NF-N)	3.2 / 3.6 / 5.9 max 6.6	46 / 98 / 481	142 ± 16	326 ± 96	4.2 ± 0.1
2 (AR-ND)					
3 (AA-ND)	1.7 / 2.4 / 3.2	47 / 157 / 179	134 ± 16	280 ± 104	4.0 ± 0.1
4 (AR-D)	2.6	156	117	312	3.8
5 (AA-DCR)					
6 (NF-ND)	2.7 / 3.2 / n.e.	76 / 100 / n.e.	136 ± 3	15 ± 16	4.1 ± 0.0
7 (AR-N+CYT)	2.7 / 3.5 / n.e.	37 / 90 / n.e.	90 ± 12	64 ± 46	4.4 ± 0.3
8 (PR-NDCR)	3.2 / 3.9 / n.e.	60 / 138 / n.e.	112 ± 8	93 ± 46	4.7 ± 0.1
9 (NF-NDCR)	1.8 / 1.9 / n.e.	45 / 66 / n.e.	132 ± 23	99 ± 55	4.4 ± 0.5

*av. 21d – Average daily values for the 21 day period before excision of leafy cuttings (Light integral DLI mol m^{-2} d^{-1}, Air humidity %, Temp °C)

** low/high/excess – Initial uniform peat characteristics: mg l^{-1} 167 NO_3-N, 75 P, 133 K, 4.1 pH, 1.83 g l^{-1} KCl, (Nutrient solutions – NL = 150 ml plant^{-1} week^{-1} supplied equal amounts of 29±4 mg P, 99±15 mg K)

a , b – a – donor plants received supplementary assimilatory light during 11 days before shoot tip excision by high-pressure sodium vapour lamps SON-T AGRO (Philips) 400W,
b – Period of yield registration was restricted to 21 days prior to the date of shoot tip excision

z – Average nitrogen supply rate provided for the total period of donor plant cultivation

n.e. – not executed during cultivation, i.e. no donor plants were treated with excess nitrogen supply

Growth of cuttings (i.e. axillar shoot tips) at differential nitrogen availability

Growth of *Petunia hybrida* donor plants was monitored on rising N fertigation levels (low, high, excess) by the cumulated number of excised shoot tips (cutting yield per donor plant) and their biomass (biomass of cuttings per donor plant) (details in methods and table of experiments above). They differed among the three nitrogen regimes, while a low N-level resulted mean reductions of both cutting yield (-20%) and biomass (-33%) when compared to the high N-level. In contrast, N-excess fertigation showed the same cutting yield as the high N-level but endured reductions of biomass (-12%). Thereby, especially the N-excess regime exceeded N uptake by donor plants which caused a sizable accumulation of nitrate-N in their root compartment (see Exp. 1 and 2 in the table before). This in turn resulted in an increased ionic strength in the soil solution as recorded via salt accumulation in the root environment of the donor plants *(Salt*^{KCl} *equivalent: N-low/-high/-excess 3.2/3.6/5.9 up to max 6.6 g KCl l*$^{-1}$*peat)* and upon further detrimental osmotic interference on the physiology of cuttings (see in the paper text chapter: Change of free proteinogenic amino acids).

Explanations of Experiment Codes

1 (NF- N)	– Nitrogen fraction analyses (NF-pools) of excised cuttings with low, high and excess nitrogen fertigation (N) treatments
6 (NF- ND)	– Nitrogen fraction analyses (NF-pools) of excised cuttings with low and high nitrogen fertigation (N) and dark exposure (D) treatments
9 (NF- NDCR)	– Nitrogen fraction analyses (NF-pools) in course of rooting (CR) under light with low and high nitrogen fertigation (N) and dark exposure (D) treatments
7 (AR- N+CYT)	– Adventitious root formation (AR) with low and high nitrogen fertigation (N) treatments and histological analyses of early cytological (CYT) events in stem base tissues
4 (AR- D)	– Adventitious root formation (AR) with dark exposure (D) treatments
2 (AR- ND)	– Adventitious root formation (AR) with low and high nitrogen fertigation (N) and dark exposure (D) treatments
3 (AA- ND)	– Amino acid analyses (AA) of excised cuttings with low, high and excess nitrogen fertigation (N) and dark exposure (D) treatments
5 (AA- DCR)	– Amino acid analyses (AA) in course of either (i) direct rooting (CR) or (ii) dark exposure (D) of cuttings or (iii) dark exposure and subsequent rooting (D+CR)
8 (PR- NDCR)	– Soluble protein analyses (PR) of excised cuttings with low and high nitrogen fertigation (N) treatments in course of either (i) direct rooting (CR) or (ii) dark exposure and subsequent rooting (D+CR)
