# Supplementary material for: Nitrogen remobilisation facilitates adventitious root formation on reversible dark-induced carbohydrate depletion in Petunia hybrida
Source: BMC Plant Biol. 2016 Oct 10;16:219. doi: 10.1186/s12870-016-0901-6 (PMC5056478; doi:10.1186/s12870-016-0901-6)
Supplement: Additional file 2: — Explanation of experimental designs for statistical analyses. (PDF 13 kb) [file 12870_2016_901_MOESM2_ESM.pdf]

Additional file 2: *Explanation of experimental designs for statistical analyses* (*Experiments 1 to 9 detailed in Additional file 1)*

Nitrogen fractions (NF-pools) were analysed in excised cuttings with low, high and excess nitrogen fertigation (N_d) alone (***Design 1*** of *Exp. 1: NF-N)*: 2(+1) N-levels x 2 excision dates from donor plants x 4 replications, n= 24) or in excised cuttings with low and high nitrogen fertigation (N_d) and dark exposure (D) treatments of 168 hpe (***Design 2*** of *Exp. 6: NF-ND)*: 2 N-levels x 2 levels of dark exposure x 4 replications, n=16).
Finally, NF were analysed in cuttings with low and high nitrogen fertigation (N) and dark exposure (D) treatments in course of rooting (CR) under light (***Design 3*** of *Exp. 9: NF- NDCR)*: 2 N-levels x 2 levels of dark exposure x 5 times post insertion in course of AR x 4 replications, n = 80).

Adventitious root formation (AR) – 384 hpe first considered low and high nitrogen fertigation (N) treatments alone (***Design 4*** of *Exp. 7: AR- N+CYT)* (I) for TRN, SRL, TRL and URC: 2 N- levels x 2 excision dates from donor plants x 4 replication plots, n=16; (II) for RNC: 2 N-levels x 2 excision dates x 10 cuttings per plot x 4 replication plots x 7 root length classes, n=1120). Further, AR was rated after dark exposure (D) treatments alone (***Design 5*** of *Exp.4: AR- D)* (I) for TRN, SRL, TRL and URC: 2 excision dates x 2 levels of dark exposure x 4 replications, n=16; (II) for RNC: 2 excision dates x 2 levels of dark exposure x 10 cuttings per plot x 4 replication plots x 7 root length classes, n=1120). Finally, the capacity of AR was reviewed for combinations of low and high nitrogen fertigation (N) with dark exposure (D) treatments (***Design 6*** of *Exp. 2: AR- ND)* for TRN, SRL, TRL and URC: 2 N-levels x 2 excision dates x 2 levels of dark exposure x 4 replications, n=32).

Early histological and cytological events (CYT) – at 72 hpin and 168 hpin in stem base tissues were included (***Design 7*** of *Exp. 7: AR-N+CYT)*: 2 N_d-levels x 2 replication plots of donor plants x 2 time points during early events of AR x 2 cuttings, n=16).

Soluble amino acids (AA) were analysed first in excised cuttings with low, high and excess nitrogen fertigation (N) and dark exposure (D) treatments (***Design 8*** of *Exp. 3: AA-ND)*: 3 N-levels x 2 levels of dark exposure x 5 cuttings per plot x 2 types of tissue (leaf and stem base) x 4 replication plots, n = 240). Then, amino acids (AA) were explored in either course of (i) direct rooting (CR) or (ii) dark exposure (D) of cuttings or (iii) dark exposure and subsequent rooting (D+CR) (***Design 9*** of *Exp. 5: AA-DCR)*: 3 types (i,ii,iii) of environment exposition x 5 times of exposition x 2 cuttings per plot x 2 types of shoot tissue x 4 replication plots n=240; this design was repeated completely at a second date – data not shown – while main results were reproduced independently).

Soluble proteins (PR) were analysed in excised cuttings with low and high nitrogen fertigation (N) treatments in course of either (i) direct rooting (CR) or (ii) dark exposure and subsequent rooting (D+CR)) (***Design 10*** of *Exp. 8: PR-NDCR)*: 2 N-levels x 2 levels of dark exposure x 5 times post insertion during AR x 2 types of shoot tissue x 4 replications, n=160).
