# Supplementary material for: Nitrogen remobilisation facilitates adventitious root formation on reversible dark-induced carbohydrate depletion in Petunia hybrida
Source: BMC Plant Biol. 2016 Oct 10;16:219. doi: 10.1186/s12870-016-0901-6 (PMC5056478; doi:10.1186/s12870-016-0901-6)
Supplement: Additional file 3: — Supplemental data of Figures 2 to 7. (PDF 31 kb) [file 12870_2016_901_MOESM3_ESM.pdf]

***<u>Additional file 3</u>**: Supplemental data of Figures 2 to 7(Experiments 1 to 9 detailed in Additional file 1)*

<u>Figure 2:</u> Accumulation of total nitrogen (N_t), allocation to NF-pools and dry mass increase during AR-formation

(Panel a) *<u>Effect of a graduated nitrogen (N_d) fertilisation</u>* to donor petunia plants (N-levels: N low, N high, N excess) on the range of total N absorption in terms of concentration (N_t in µmol N g^{-1} DM) and N_t allocation to selected fractionated nitrogen pools (NF-pools) in whole cuttings at severance in (*<u>Exp. 1: NF-N)</u>*. Regression relationships (y = a + b*x, y = c*exp(dx) for n=24 with $p<0.05$ between N_t (x) ($2800 < x < 5300$) and NF (y) with linear correlation coefficients (r) and regression parameters (a,b,c,d) for nitrate- (r = 0.91, a = -593.57, b = 0.2889), amide- (r = 0.88; c = 0.0002, d = 0.0029), amino- (r = 0.74, a = 334.38, b = 0.0764), insoluble protein-NF (r = 0.96, a = 1382.50, b = 0.3132) and the sum of NF (r = 0.99; a = 480.68, b = 0.8659).

(Panel b) *<u>Dry mass accumulation</u>* in petunia shoot tips during 168h rooting under light (hpin = time post insertion). Cuttings were grown at two N availability levels (N low; N high) which resulted two N_t absorption levels ($N_{t\text{-}Nlow}$ = 2900; $N_{t\text{-}Nhigh}$ = 3500 µmol N g^{-1} DM). After excision from donor plants, cuttings were rooted either directly (no dark – circle symbols) or received a 168h pre-rooting dark exposition (hde - squared symbols) at 10°C. Vertical bars represent 0.95 confidence intervals of the least squares mean values and different lower-case letters indicate significant differences in datum points in (*<u>Exp. 9: NF-NDCR)</u>*. A three factorial ANOVA revealed significant main and interaction effects for levels of N fertilization: $F(1,36)=11.246$, $p<0.005$; dark exposure (hde): $F(1,36)=21.785$, $p<0.00005$; time post insertion in course of AR formation (hpin): $F(1,36)=97.047$, $p<0.00001$ and hde x hpin: ($F(2,36)=5.46$, $p<0.01$), respectively.

Details of experimental designs for regression analyses and factorial ANOVA with (**Panel a** and **b**) are in methods and additional files 1 and 2.

<u>Figure 3:</u> Adventitious root formation 384 hours post excision (hpe) with cuttings of *Petunia hybrida*

(Panel a) *<u>N availability to donor plants (N_d-levels):</u>* Effect of two nitrogen (N_d) fertilization levels to donor plants on the number of roots developed per root length class (RNC) per single cutting at 384 hpe; Two Levels of N availability to donor plants: (Circles connected with full lines) N low providing low total N absorption of 2575 µmol N_t g^{-1} DM of whole cuttings and a total root number (TRN) of 0.83 $cutting^{-1}$; (Squares connected with intermittent lines) N high showing high total N absorption of 3629 µmol N_t g^{-1} DM and a TRN of 13.84 $cutting^{-1}$. Actual effect on RNC - rating 384hpe - with n=1120: $F(6, 1092)=79.416$, $p<0.0001$ (*<u>Exp.7: AR-N+CYT)</u>*.

(Panel b) *<u>Pre-exposure to dark:</u>* Effect of a pre-exposure to dark on root number per root length class (RNC): (Circles connected with full lines) Rating 384h after excision and immediate perlite insertion with a total RN of 19.9 $cutting^{-1}$; (Squares connected with intermittent lines) Rating 384h after excision including 168h pre-exposure to dark followed by 216h perlite insertion and a total RN of 26.8 $cutting^{-1}$ (*<u>Exp.4: AR-</u>*

D). Actual effect on RNC - rating 384hpe - with n=1120: $F(6, 1092)=40.873$, $p<0.0001$. Cuttings of both treatments grew with the same adequate N fertilisation to donor plants delivered by weekly fertigations of 0.2% Hakaphos spezial (N availability: 39 mg 100 g^{-1} dry substrate). At excision, those cuttings showed high total soluble amino acid concentrations of 7 to 9 and 10 to 15 µmol g^{-1} FM in leaf and basal stem tissues, respectively.

Vertical bars represent 0.95 confidence intervals of the least squares mean values and different lower-case letters indicate significant differences in datum points. While vertical bars remain invisible the symbol size surpassed the confidence interval in (**Panel a** and **b**).

(Panels c and d) ***N availability (N_d) to donor plants x Pre-exposure to dark:*** Root development in response to interaction effects of two N supply levels to donor plants (N absorption of whole cuttings: N low = 3112 µmol N_t and N high = 4034 µmol N_t g^{-1} DM) and a 168h pre-exposure to dark at 10°C observed per singel cutting with total root number (TRN) in (**Panel c**) and total root length (TRL) in (**Panel d**). (Rating 384h after excision either with immediate perlite insertion (gray columns) or with 168h pre-exposure to dark followed by 216h perlite insertion (black columns) (**Panels c and d**, *Exp. 2: AR-ND*). Actual ineraction effects with n=32 in (**Panel c**) for TRN: $F(1, 24)=11.675$, $p=0.00226$ and in (**Panel d**) for TRL: $F(1, 24)=7.6374$, $p=0.01080$.

(Panels e, f, g and h) ***Regression relationships*** between N_t contents (x) ($2400 < x < 3900$) and (y) as total root length (TRL in **Panel e**, r=0.82), as total root number (TRN in **Panel f**, r=0.88), as single root length (SRL in **Panel g**, r=0.84) and as percentage of unrooted cuttings determined 384 hpe (URC in **Panel h**, r=0.89) (N fertilization to donor plants: N_d low - Squares and N_d high - Circles) (**Panels e to h**, *Exp. 7: AR-N+CYT*).
Regression relationships ($y = a + b*x$, n=16, $p<0.05$) with linear correlation coefficients (r) and regression parameters (a,b) for total root length (**Panel e**: TRL, r = 0.82, a = -38.0755, b = 0.0153), total root number (**Panel f**: TRN, r = 0.88; a = -27.5128, b = 0.0112), single root length (**Panel g**: SRL, r = 0.84, a = -1.3888, b = 0.0007) and cuttings unrooted (**Panel h**: URC, r = 0.89, a = 220.1195, b = - 0.0597).

Figure 4: Change of total nitrogen (N_t) and NF-pools with two donor plant N_d levels, dark exposure and AR formation of cuttings

(Panels a to j) Effects of (1) graduated nitrogen (N_d) fertilisation to donor petunia plants (N_d-levels: N low, N high), (2) dark cold exposures of excised cuttings (168hde=168hpe, 10°C) and (3) times post insertion for AR formation under diurnal light (0, 6, 24, 72, 168hpin), when assayed in two experiments (**Panels a to e**, *Exp. 6: NF-ND* and **Panels f to j**, *Exp. 9: NF-NDCR*) with whole cuttings for total N absorption (N_t) in (**Panels a and f**) and related N_t allocations to the specific nitrogen fractions (NF) as Amino-N in (**Panels b and g**), Nitrate-N in (**Panels c and h**), Protein-N in (**Panels d and i**) and Amide-N in (**Panels e and j**). Vertical bars represent 0.95 confidence intervals of the least squares mean values.

Multifactorial ANOVA's revealed significant main and interaction effects **with N_t and NF**, respectively:

N_d-levels: in (**Panel a**): F(1, 12)=239.08, p<0.00001; in (**Panel c**): F(1, 12)=81.748, p<0.00001; in (**Panel d**): F(1, 12)=337.53, p=0.00001; in (**Panel f**): F(1, 60)=371.07, p<0.0001; in (**Panel g**): F(1, 60)=85.892, p<0.00001; in (**Panel i**): F(1, 60)=171.57, p<0,0001; in (**Panel j**): F(1, 60)=10,113, p<0.005

Dark exposure: in (**Panel d**): F(1, 12)=137.93, p<0.00001

Time post insertion: in (**Panel f**): F(4, 60)=132.90, p<0.0001

N_d-levels x dark exposure: in (**Panel b**): F(1, 12)=5.1960, p<0.05; in (**Panel e**): F(1, 12)=14.390, p<0.005

N_d-levels x time post insertion: in (**Panel h**): F(4, 60)=4.1150, p=0.01

Dark exposure x time post insertion: in (**Panel g**): F(4, 60)=5.7968, p<0.001; in (**Panel i**): F(4, 60)=7.5715, p<0.0001; in (**Panel j**): F(4, 60)=18.308, p<0.00001, respectively.

Figure 5: Change of proteinogenic amino acids with graduated donor plant N_d fertilisation and dark exposure of cuttings

(Panels a to f) Multifactorial ANOVA’s revealed significant main and interaction effects of (1) N_d-levels, (2) dark exposure and (3) tissue type on total amino acids in (**Panel a**), glutamate in (**Panel b**), aspartate in (**Panel c**), arginine in (**Panel d**), glutamine in (**Panel e**) and asparagine in (**Panel f**) in *(Exp. 3: AA-ND)*. Vertical bars represent 0.95 confidence intervals of the least squares (**Panel a, b, c, d**) and weighted (**Panel e, f**) mean values:

N_d-levels x dark exposure x tissue type: (1 x 2 x 3) in (**Panel a**) F(2, 228)=15.446, p<0.00001; (**Panel c**) F(2, 228)=3.8547, p<0.05; (**Panel d**) F(2, 228)=46.510, p<0.0001; (**Panel e**) F(2, 228)= 3.4120, p<0.05; (**Panel f**) F(2, 228)= 11.966, p<0.00001

N_d-levels x tissue type: (1 x 3) in (**Panel b**) F(2, 228)=13.658, p<0.00001

Dark exposure: (2) in (**Panel b**) (F(2, 228)=34.137, p<0.000001, respectively.

Figure 6: Course of total and single amino acids in leaf and stem base exposed to three environment conditions

(Panels a to r) Multifactorial ANOVA’s revealed significant interaction of (1) environment condition, (2) exposition time within a specific environment (0, 6, 24, 72, 168 hpin or hde, respectively) when assayed (3) in leaf or stem base tissue of excised cuttings for levels of amino acids (AA) in (**Panel a, b, c**), glutamine in (**Panel d, e, f**), asparagine in (**Panel g, h, i**), arginine in (**Panel j, k, l**), glutamate in (**Panel m, n, o**) and aspartate in (**Panel p, q, r**) *(Exp. 5: AA-DCR)*. Vertical bars represent 0.95 confidence intervals of the least squares mean values:

Environment condition x exposition time x tissue type: (1 x 2 x 3) in (**Panels a-b- c**) F(8, 210)=6.6655, p<0.00001; (**Panels d-e-f**) F(8, 210)=2.3457, p<0.05; (**Panels g-h-i**) F(8, 210)=5.4262, p<0.00001; (**Panels j-k-l**) F(8, 210)=48.142, p<0.0001; (**Panels m-n-o**) F(8, 210)=24.428, p<0,0001; (**Panels p-q-r**) F(8, 210)=11.680, p<0.00001, respectively.

The three distinct environment conditions regarded (i) Rooting in (**Panels a, d, g, j, m, p**) with immediate perlite insertion for AR formation under light (hpin=hpe), (ii) Dark exposition in (**Panels b, e, h, k, n, q**) with dark and cold exposure at 10°C (hde=hpe) and (iii) Rooting after dark in (**Panels c, f, i, l, o, r**) with insertion for AR formation under light when cuttings had received a pre-rooting dark and cold exposure for 168h at 10°C (hpin+168hde=hpe).

Cuttings of all treatments grew with the same adequate N_d fertilisation to donor plants delivered by weekly fertigations of 0.2% Hakaphos spezial (N availability: 39 mg 100 g^{-1} dry substrate).

Figure 7: Course of soluble protein in leaf and stem base of cuttings

(Panels a to d) Multifactorial ANOVA's revealed significant interaction effects with **soluble proteins** for a (1) graduated nitrogen (N_d) fertilisation to donor petunia plants (N_d-levels: N-low; N-high), (2) dark and cold exposure of excised cuttings for 168h at 10°C before insertion for AR formation and (3) exposition time after perlite insertion under diurnal light for AR formation when assayed (4) in stem base (**Panel a**) or leaf (**Panel b**) tissue for levels of soluble proteins *(Exp. 8: PR-NDCR)*.

N_d-level x dark exposure x exposition time: (1 x 2 x 3) with (**Panels c, d**) $F(4, 120)=2.8788$, $p<0.05$ and

Dark exposure x exposition time x tissue type: (2 x 3 x 4) in (**Panels a, b**) $F(4, 120)=6.0865$, $p<0.0005$, respectively.

The two N_d fertilization levels to donor plants resulted in different N_t concentrations in cuttings with N_t-N low = 3190 in (**Panel c**) and N_t-N high = 3850 in (**Panel d**) in µmol N g^{-1} DM, respectively. Vertical bars represent 0.95 confidence intervals of the least squares mean values.
